# Metagenomic and Metabolomic Profiling Reveals the Differences of Flavor Quality between *Hongqu* Rice Wines Fermented with *Gutian* Qu and *Wuyi* Qu

**DOI:** 10.3390/foods13193114

**Published:** 2024-09-29

**Authors:** Zihua Liang, Shiyun Chen, Hao Wang, Qi Wu, Weiling Guo, Li Ni, Xucong Lv

**Affiliations:** 1 Institute of Food Science and Technology, College of Biological Science and Engineering, Fuzhou University, Fuzhou 350108, China; 220820089@fzu.edu.cn (Z.L.); 220820074@fzu.edu.cn (S.C.); 230827089@fzu.edu.cn (H.W.); 200827089@fzu.edu.cn (Q.W.); weilguo@fzu.edu.cn (W.G.); nili@fzu.edu.cn (L.N.); 2 Food Nutrition and Health Research Center, College of Advanced Manufacturing, Fuzhou University, Jinjiang 362200, China

**Keywords:** *Hongqu* rice wine, metagenomics, metabolomics, volatile flavor components, biogenic amines

## Abstract

*Jiuqu* (starter) makes an important contribution to the formation of the flavor characteristics of *Hongqu* rice wine (HQW). *Gutian* Qu (GTQ) and *Wuyi* Qu (WYQ) are two kinds of *Jiuqu* commonly used in HQW brewing, but the comparison of the two kinds of HQW is still insufficient at present. The objective of this study was to compare the dynamic changes of amino acids (AAs), higher alcohols (HAs), bioamines (BAs), volatile flavor compounds (VFCs), and microbial communities in HQW fermentation, with GTQ and WYQ as starter. This study used an automatic amino acid analyzer, GC, HPLC, and GC-MS to detect AAs, HAs, Bas, and VFCs during fermentation; metagenomic sequencing technology was used to elucidate the microbial community and its functional characteristics. The results showed that the contents of AAs and HAs in HQW brewed with WYQ (WYW) were significantly higher than those in HQW brewed with GTQ (GTW). On the contrary, the majority of BAs in GTW were significantly higher than those in WYW. The composition of VFCs in WYW and GTW were obviously different, as most of the VFCs were notably enriched in WYW, while ethyl caproate, isoamyl acetate, ethyl heptanoate, ethyl nonanoate, 1-decanol, citronellol, phenethyl acetate, and hexanoic acid were more abundant in GTW. *Burkholderia gladioli*, *Pantoea dispersa*, *Weissella cibaria*, *Monascus purpureus*, and *Saccharomyces cerevisiae* were the predominant microbial populations in GTW brewing at the species level, while *Sphingomonas* sp., *Kosakonia cowanii*, *Enterobacter asburiae*, *Leuconostoc lactis*, *Aspergillus niger*, and *Saccharomyces cerevisiae* were the dominant microbial species in WYW brewing. The abundance of functional genes involved in BAs biosynthesis were much higher in GTW brewing, while the abundance of functional genes related to the metabolism of characteristic VFCs were much higher in WYW brewing. Collectively, these findings provided evidence for elucidating the effects of *Jiuqu* and microbial communities on HQW flavor quality, and laid a solid foundation for the improvement of HQW flavor quality.

## 1. Introduction

Huangjiu holds an important position in Chinese historical civilization as a traditional fermented alcoholic beverage [1]. Among the varieties of Huangjiu, *Hongqu* rice wine (HQW) stands out for its bright crimson color, pleasant sweetness, potential health benefits, and has a brewing history of at least 1000 years in China [2]. Compared to the strict sterile environment commonly required by most modern fermented food processing industries, the brewing process of HQW is carried out in a non-sterile environment [3]. Traditional brewing processes and non-sterile environments lead to complex microbial evolution and metabolic interactions, ultimately endowing HQW with unique flavor quality, but at the same time bringing certain food safety risks [1]. In addition to the influence of the fermentation environment on microbial communities, the type of *Hongqu* is also crucial to the formation of microflora and flavor quality in HQW brewing. Currently, there are two types of *Hongqu* commonly used in HQW brewing industry, namely *Gutian* Qu (GTQ) and *Wuyi* Qu (WYQ) [4]. Among them, GTQ is prevalent in Gutian County of Fujian Province in China (the famous birthplace of *Hongqu*), and its dominant microorganism is mainly *Monascus* spp. [5]. In contrast, WYQ exhibits a more complex and diverse range of microorganisms, as it is usually prepared through solid-state fermentation of rice in a non-sterile environment, using a traditional process of inoculating Quqing (also known as black skin-Qu, abundant in *Monascus*, *Rhizopus*, and *Aspergillus*, etc.) [6]. Our two previous studies, based on amplicon high-throughput sequencing, have clarified the clear differences in microbial composition between GTQ and WYQ [4,6], which would inevitably result in a difference in microbial communities and metabolomic profiles in HQW brewing, and then affect the ultimate flavor quality of HQW. Nevertheless, limited research has been undertaken to compare the dynamics of amino acids (AAs), higher alcohols (HAs), biogenic amines (BAs), volatile flavor compounds (VFCs), microbial composition, and functional genes involved in AA, HA, and BA metabolism in rice wine brewing, with GTQ and WYQ as fermentation starters.

Flavor is one of the important indexes to measure the acceptability of rice wine. The dynamic change of flavor compounds during RW fermentation is essentially the change of microbial metabolism level, and the unique microbial metabolism of RW is the key to the formation of its unique flavor [7]. The main aroma components of RW are esters, alcohols, and phenols. For example, esters are mainly synthesized by esterification enzymes produced by microorganisms; alcohols are mainly synthesized due to the yeast in the alcohol fermentation process through the glycolysis pathway, which converts sugars into alcohols [3]. Therefore, a deeper investigation is warranted to elucidate the effects of microbial communities and their metabolites on RW flavor quality. However, it is also inevitable that the metabolism of microorganisms in the non-sterile open winemaking process will produce some potentially harmful substances that will affect the safety of the RW. HAs, as one of the flavor compounds in alcoholic beverages, contribute greatly to the flavor style of RW. Moderate amounts of HAs can give RW special sensory characteristics such as richness, aroma, and mellow sweetness. Unfortunately, high levels of HAs (greater than 300 mg/L) have been shown to negatively affect the flavor quality and post-drinking comfort of alcoholic beverages, with adverse effects such as “headaches” and “nausea” after drinking [8]. BAs, a potential hazard, are also reported to be prevalent in fermented foods. They are formed by microorganisms with amino acid decarboxylase activity acting on corresponding amino acids to decarboxylate them [9]. One study has shown that, when drinking BA-rich RW, it is more likely to cause negative physiological reactions such as headache, palpitations, vomiting, or dilated pupils [10]. Furthermore, alcohol significantly inhibits the catalytic activity of amine oxidase, so the restriction of BAs in alcoholic beverages should be more stringent [11]. Unfortunately, previous studies on HQW brewed by GTQ and WYQ have not explored the formation pathways of harmful substances, such as HAs and BAs, or the regulatory effects microbial species have on them during the brewing process. Therefore, elucidating the potential association between microbiota and harmful metabolites is of great significance for improving the flavor quality and safety of HQW.

In our previous studies, amplicon sequencing based on 16S/ITS partial hypervariable regions was conducted to initially reveal the differences in microbial communities in WYQ and GTQ, and 16S/ITS full-length amplicon sequencing was also used to analyze the differences in microbial community succession during WYW and GTW brewing [4,6]. However, it is well known that 16S/ITS amplicon sequencing technology has certain defects in the analysis of microbial communities in fermented foods, mainly being unable to analyze the composition and metabolic function of microorganisms at the species and gene level [12,13]. Consequently, these recognized constraints in amplicon-based microbial community analysis have raised concerns about the accuracy and reproducibility of phylogenetic marker studies. Metagenomic sequencing is the sequencing of the sum of all microbial DNA in a sample, which can give an overview of the composition, structure, and potential functions of the microbial community [14]. Indeed, metagenomics has significantly advanced our comprehension of the microbiology of various fermented foods and holds promising potential for applications in ensuring food quality and safety [15]. Thus, metagenomic sequencing can be effectively utilized to functionally explore potential microbes involved in HQW fermentation, gain deeper insights into the core microbial differences between GTW and WYW, and regulate core functional microbes to mitigate the formation of harmful metabolites and enhance flavor quality.

In this study, we compared the dynamics of AAs, HAs, BAs, and VFCs during HQW brewing fermented with GTQ and WYQ. Secondly, metagenomic sequencing technology was utilized to compare the microbial communities of WYW and GTW brewing at the species level. Finally, the microbial species and functional genes involved in the metabolism of AAs, HAs, BAs, and characteristic VFCs were examined through bioinformatics analysis. The purpose of this work is to further explore the correlation between microbes and flavor quality formation in HQW brewing, to better understand the key microorganisms and their contributions to HQW flavor quality, and ultimately to promote the controllability of HQW brewing.

## 2. Materials and Methods

### 2.1. Sample Collection

*Gutian* Qu (GTQ) and *Wuyi* Qu (WYQ) used for HQW fermentation were obtained from the wine factories in Gutian and Anxi county of Fujian Province of China, respectively. Before initiating the brewing, the wine tanks were disinfected with 100 °C boiled water, and the glutinous rice was washed, soaked in water at room temperature overnight, and then steamed at 100 °C for 45 min. After being cooled to ambient temperature, the steamed rice was transferred to wine tanks, then mixed with 10% *Hongqu* and 150% sterilized water. The wine tanks were wrapped with eight layers of gauze for fermentation (18 °C, 10 days) with daily manual stirring and mixing. From the tenth day of fermentation, the eight layers of gauze were substituted with plastic film to facilitate anaerobic fermentation (18 °C for 35 days). The fermentation process is the same for both types of *Jiuqu*.

Three parallel independent experiments were conducted for GTW and WYW brewing. Fermentation samples were collected at different fermentation stages (1, 2, 3, 5, 7, 10, 20, 30, and 45 days) of GTW and WYW brewing. The fermentation samples were filtered through 4 layers of gauze, and the filtrates were further centrifuged (10,000× *g*) at 4 °C for 10 min to obtain the supernatants and the sediments. The supernatant was diluted 10-fold and used for the detection of physicochemical properties, AAs, BAs, Has, and VFCs; the sediments were used for metagenomic sequencing. Due to the large number of uncontrollable factors in simulating traditional fermentation, the experimental conditions of each group were controlled to be consistent, the tests were repeated three times in each group under the same conditions, and the arithmetic average value was taken as the final result.

### 2.2. Physiochemical Parameter Determination

The ethanol contents of HQW fermentation samples were determined by gas chromatography. In short, the sample was filtered with a 0.22 μm aqueous filter membrane, the filtrate was placed in the sample vial, tested with a gas chromatogram equipped with a hydrogen flame ionization detector and an Agilent J&W CP-Wax 57 CB capillary column (30 m × 0.25 mm × 0.25 μm), an ethanol standard curve was made, and quantitative analysis was performed according to the standard curve. The glucose standard curve was prepared and the content of reducing sugar was determined by 3, 5-dinitrosalicylic acid method at 540 nm. The total acids content was determined according to the Chinese official method (GB/T 13662-2018 rice wine). The above specific operation methods are described in our previous study [16].

### 2.3. Determination of Free Amino Acids (AAs)

The composition and content of free AAs in wine mash obtained from GTW and WYW brewing were measured by L-8900 automatic amino acid analyzer (Hitachi, Tokyo, Japan) according to a previous report [17]. The contents of different AAs in fermented samples of GTW and WYW were calculated by peak area method through the plotting curve of the standards of different AAs.

### 2.4. Determination of Higher Alcohols (HAs)

The fermentation samples were filtered with a 0.22 μm microporous membrane, and the filtrate were put into a 2.0 mL sample bottle. The contents of HAs were determined by gas chromatography. The specific operation methods and instrument parameters were referred to the previously reported literature in detail [16].

### 2.5. Determination of Biogenic Amines (BAs)

The contents of BAs in fermentation samples from GTW and WYW brewing were determined by HPLC. Briefly, 1 mL sample solution and 50 mg polyvinylpyridone K30 were added into a 2 mL centrifuge tube, thoroughly mixed, then shaken at 25 °C and 500 rpm for 15 min. The filtrate was obtained by passing the sample through Whatman qualitative No. 4 filter paper. Subsequently, 100 μL of filtrate was taken and 400 μL of sodium bicarbonate buffer, 300 μL of acetone, and 200 μL of salvinorin chloride solution were added. The mixture was vortexed for 30 s and incubated in a water bath at 60 °C for 1 h. BAs were quantitatively and qualitatively analyzed by using an Agilent 1260 Infinity II HPLC instrument (Agilent Technologies, Little Falls, DE, USA) equipped with an UV detector and Accucore™ C18 HPLC column (4.6 × 250 mm, 5 μm, Thermo Scientific, Waltham, MA, USA). The mobile phase and detection parameters used in the HPLC detection of BAs in fermented samples were referred to our previous study [18].

### 2.6. Determination of Volatile Flavor Compounds (VFCs)

VFCs were extracted by HS-SPME and detected by GC-MS (7890A GC-5977A MS system, Agilent Technologies, Little Falls, DE, USA), equipped with Agilent HP-INNOWAX capillary chromatography column (30 m × 0.25 mm, 0.25 μm film thickness). According to the peak area of the internal standard compound (2-octanol, 10 mg/L) and the standard curve of the reference compounds, the concentrations of VFCs were quantitatively calculated. Based on the ratio of the concentrations of VFCs to their odor thresholds, the odor activity values (OAVs) of the selected VFCs were calculated. All analyses were repeated for at least three replicates. For specific operating methods and instrument conditions, refer to previous literature [4].

### 2.7. Microbial DNA Extraction and Metagenomic Analysis

Microbial DNA was extracted from a fermenting mash of GTW and WYW brewing collected at days 2, 5, 10, 20, and 30 using the CTAB-based method [19]. Subsequently, the microbial DNA was sent to Illumina NovaSeq Xten (Illumina Inc., San Diego, CA, USA) of Majorbio Bio-Pharm Technology Co., Ltd. (Shanghai, China) for metagenomic sequencing. First of all, quality control should be carried out on the original sequencing data to obtain high-quality clean data and ensure the accuracy of subsequent analysis results. Quality control and assembly of sequences were performed on the free online platform of Majorbio Cloud Platform (http://www.majorbio.com, accessed on 11 April 2022). Briefly, the paired-end Illumina reads were trimmed of adaptors, and low-quality reads (length < 50 bp or with a quality value <20) were removed by fastp (version 0.20.1). The paired-end Illumina reads were quality-filtered, trimmed, and screened using SeqPrep (https://github.com/jstjohn/SeqPrep, accessed on 11 April 2022) and Sickle (https://github.com/najoshi/sickle, version 1.33, accessed on 11 April 2022). Subsequent metagenomic sequencing and annotation referred to the method described in a previous study [18,20]. All raw data from metagenomic sequencing has been uploaded to NCBI’s SRA database under the registration number PRJNA1119967.

### 2.8. Multivariate Statistical Analysis and Visualization

The test results of the physicochemical properties in the brewing process were expressed as mean ± standard deviation, and one-way ANOVA were performed by GraphPad Prism (version 9.5). A heatmap and bubble chart were drawn by R software (version 3.3.3) to visualize the composition of VFCs and the abundance of functional genes. Principal component analysis (PCA) was performed using SIMCA software (version 14.1) to characterize the dynamics of VFCs. Differences in microbial taxonomic abundance between GTW and WYW at the species level were analyzed using STAMP software (version 2.1.3) and based on the Welch’s *t*-test.

## 3. Results and Discussion

### 3.1. The Dynamics of Physicochemical Parameters during GTW and WYW Brewing

**Figure 1:**
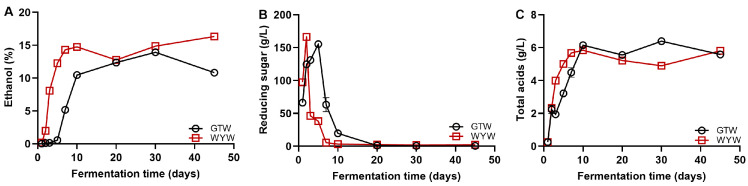
The dynamic change of physicochemical parameters including ethanol (**A**), reducing sugar (**B**), and total acidity (**C**) during GTW and WYW brewing.

The detection of physicochemical parameters is helpful when monitoring whether the fermentation process is normal and provides a scientific basis for substance transformation in HQW; therefore, the ethanol, reducing sugar, and acidity in HQW were monitored [21]. As depicted in Figure 1, significant changes and differences were observed in all three physicochemical parameters mentioned above during GTW and WYW brewing. The ethanol content of the two kinds of HQW increased during the whole fermentation process, WYW reached a peak of 16.32% (*v*/*v*) on day 45 and GTW reached a peak of 14.46% (*v*/*v*) on day 30 (Figure 1A). In the late fermentation period, the ethanol content of GTW and WYW remained relatively constant and almost reached a plateau, which was mainly attributed to nutrient depletion, as well as ethanol and acid stress resulting in yeast growth and metabolism limitation [6]. The reducing sugar of WYW and GTW accumulated rapidly at the beginning of fermentation, reaching peaks of 166.22 ± 4.52 g/L and 155.17 ± 1.78 g/L on the 2nd and 5th days of brewing, respectively, and was then rapidly consumed (Figure 1B). It is generally believed that the starch in grains is hydrolyzed into reducing sugars through the action of α-amylase and glucoamylase secreted by molds. Subsequently, reducing sugars are further consumed by yeasts, molds, and lactic acid bacteria, producing alcohol, lactic acid, and other metabolites [22,23]. Organic acids are important contributors to the flavor of RW [24]. The acidity of GTW and WYW increased dramatically 10 days before fermentation, tended to stabilize in the middle and later stages of fermentation, and maintained around 6 g/L on the 45th day of fermentation (Figure 1C). The change in acidity during fermentation can be attributed to two factors: firstly, in the early stage of fermentation, rich reducing sugars and low alcohol content contribute to the proliferation and acid production of lactic acid bacteria; secondly, the proliferation of yeast during the fermentation process produces ethanol, thereby inhibiting the activity of acid-producing microorganisms [25].

### 3.2. The Dynamics of AAs during GTW and WYW Brewing

**Figure 2:**
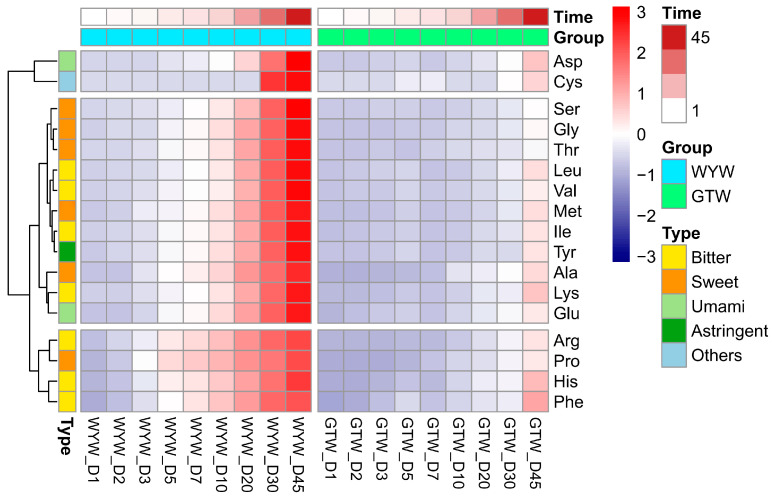
The dynamic change of amino acids during GTW and WYW brewing.

As the national wine of China, rice wine is also called “liquid cake” because of its rich amino acid content and other nutrients. AAs in rice wine are mainly produced by microorganisms through the secretion of proteases to degrade the proteins in the raw materials, while some amino acids may also be produced by yeast autolytic degradation [7]. AAs and their derivatives are the main flavor substances in many fermented foods, and their taste characteristics can be classified as umami (including Asp and Glu), sweet (including Ala, Gly, Ser, Pro, Met and Thr), bitter (including Arg, His, Ile, Leu, Lys, Phe and Val), and astringent (Tyr) [26]. As shown in Figure 2, during the brewing process of GTW and WYW, the content of almost all AAs showed a gradual increase trend, but the increase trend of most AAs in GTW was relatively slow. In the middle and late stages of fermentation, the contents of various amino acids were generally lower in GTW than in WYW.

### 3.3. The Dynamics of HAs during GTW and WYW Brewing

**Figure 3:**
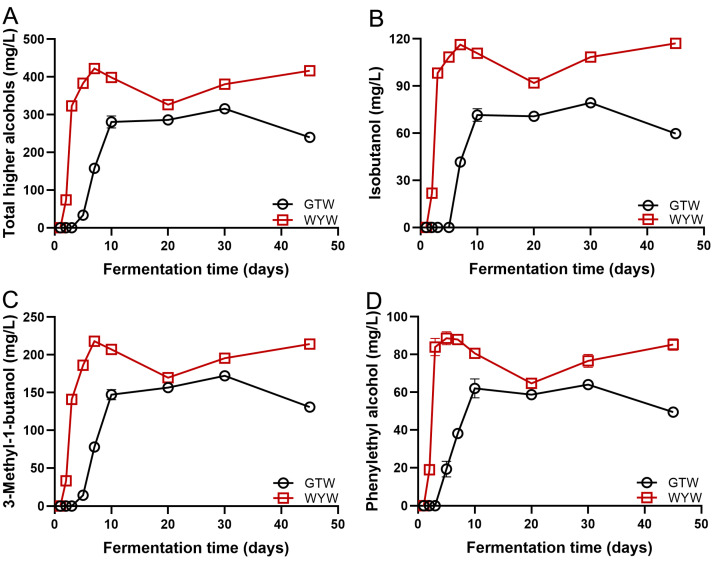
The dynamic change of HAs including total HAs (**A**), isobutanol (**B**), 3-methyl-1-butanol (**C**), and phenethyl alcohol (**D**) during GTW and WYW brewing.

Moderate levels of HAs enhance the flavor quality of wine, while excessive HAs can result in pungent and off-putting odors [27]. It has also been demonstrated that the uncomfortable symptoms after drinking rice wine are mainly caused by the high content of HAs [10]. As depicted in Figure 3A, the growth trend of HAs in the WYW and GTW brewing processes is similar, that is, the HAs content in both WYW and GTW rapidly increases during the initial stage of fermentation and continues until the 10th day of fermentation. Subsequently, it tends to stabilize. On the 20th day of WYW fermentation and the 45th day of GTW fermentation, we observed a certain degree of decrease in HAs content. This phenomenon may be attributed to esterification reactions occurring between specific HAs and organic acids [28,29]. Late in fermentation, the accumulated HAs content in WYW (416.56 ± 8.45 mg/L) was significantly higher than that in GTW (239.93 ± 2.50 mg/L). Previous study has emphasized that isobutanol, 3-methyl-1-butanol, and phenylethanol in HAs are the main factors affecting the level of comfort experienced after drinking Huangjiu [10]. In this study, compared with GTW, the levels of isobutanol, 3-methyl-1-butanol, and phenylethanol in WYW were relatively higher (Figure 3B–D). At the end of fermentation, the concentrations of isobutanol, 3-methyl-1-butanol, phenylethanol, and total HAs content in WYW was 1.96, 1.64, 1.72, and 1.74 times higher than those in GTW, respectively. Therefore, we conclude that the lower content of HAs in GTW provides a positive contribution to its post-drinking comfort.

### 3.4. The Dynamics of BAs during GTW and WYW Brewing

**Figure 4:**
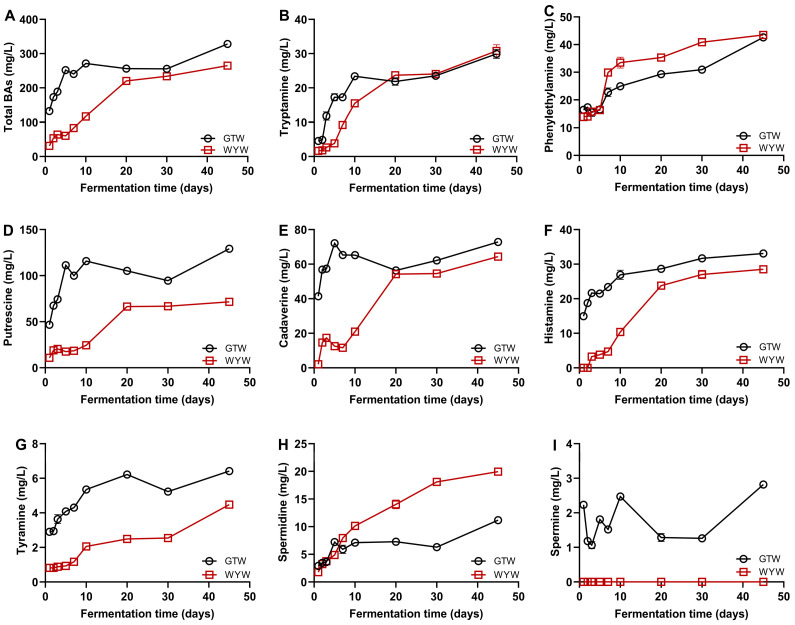
The dynamic change of BAs including total BAs (**A**), tryptamine (**B**), phenylethylamine (**C**), putrescine (**D**), cadaverine (**E**), histamine (**F**), tyramine (**G**), spermidine (**H**), and spermine (**I**) during GTW and WYW brewing.

BAs are potential hazards commonly found in fermented foods such as rice wine, and their high level of accumulation in the body may lead to physiological toxicity and even harm health [30,31]. Therefore, it is necessary to conduct in-depth exploration of the dynamic characteristics of BAs during GTW and WYW brewing. As shown in Figure 4, a total of eight BAs were identified in GTW and WYW brewing. Among them, putrescine and cadaverine were the main BAs in GTW and WYW brewing, which were mainly produced during the initial stage of fermentation. The contents of BAs in brewing exhibited a sharp increase in the early stages of fermentation, followed by a gradual upward trend. At the end of fermentation, the concentration of BAs in GTW (328.07 ± 1.88 mg/L) was significantly higher than that in WYW (264.91 ± 4.55 mg/L). Interestingly, the production of putrescine, cademine, histamine, tyramine, and spermine was consistently lower than that of GTW at various stages of the brewing process in WYW. Relevant literature has shown that the production and degradation of BAs during HQW brewing is closely related to amino acid decarboxylase and amine oxidase produced by microorganisms in the brewing system [18]. However, there is a complex interaction between the microorganisms that produce and degrade BAs, and how their quantity, species, and metabolic activity affect the production and degradation of BAs to some extent. The difference in microbial composition during the brewing process of GTW and WYW is the main reason for their immensely different BA contents. Therefore, examining the microbial community dynamics and their relationship with BA production is crucial for a deeper understanding of the mechanism behind BAs formation in HQW brewing.

### 3.5. The Dynamics of VFCs during GTW and WYW Brewing

**Figure 5:**
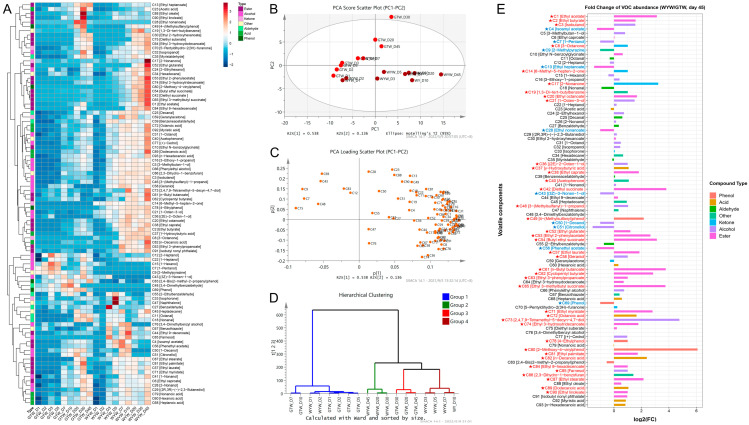
The dynamic change of VFCs during GTW and WYW brewing. Heatmap (**A**), score scatter plot (**B**), loading scatter plot (**C**) of PCA, hierarchical clustering diagram (**D**), and fold change chart (**E**). VFCs marked with a red asterisk indicate that the substances is riched in WYW; VFCs marked with a blue asterisk indicate that the substances is riched in GTW.

VFCs are an important part of RW in the fermentation process and are also an indispensable means to evaluate the fermentation technology of RW. Exploring the production rule of VFCs in the fermentation of RW is helpful to scientifically understand and improve the sensory quality of RW [32]. In this study, HS-SPME/GC-MS was employed to determine the composition and contents of VFCs in the fermentation process of GTW and WYW. As shown in Figure 5A, a total of 93 VFCs were detected in the brewing samples of GTW and WYW. Among them, esters and alcohols are the main components of the volatile flavor, and they have pleasant floral and fruity notes, which gives RW a mellow and sweet body [33]. It can be seen from the heatmap that most of the VFCs in GTW and WYW were almost undetectable at the beginning of brewing, however, most of them tended to increase gradually as the fermentation progressed. Further, there were obvious differences between GTW and WYW in the composition and content of VFCs, which we investigated by means of principal component analysis (PCA). As shown in Figure 5B, the first principal component (PC1) accounted for 53.8% of the total variation, and PC2 accounted for 13.6% of the total variation. The flavor substances of the two HQW in the early stage of brewing were generally located on the left side of the x axis of the PCA score scatter plot. As the fermentation progressed, the sample points of GTW and WYW on the PCA score scatter plot increasingly diverged, indicating an increasing disparity in the VFCs between GTW and WYW. Notably, throughout the fermentation process, WYW samples exhibited an obviously faster migration rate and greater span compared to GTW samples, suggesting a higher fermentation rate and more pronounced change in volatile profile for WYW brewing. The PCA loading plot further elucidated the characteristic VFCs of the respective fermentation samples (Figure 5C). The results revealed that both GTW and WYW exhibited minimal presence of VFCs in the initial stages of fermentation. In the later stages of fermentation, the volatile flavor profile of WYW was mainly characterized by compounds such as 2-nonanone [C17], ethyl 9-decenoate [C44], naphthalene [C47], phenethyl acetate [C56], geraniol [C58], benzothiazole [C67], and 3,4-dimethylbenzyl alcohol [C76], while the volatile flavor profile of of GTW was mainly characterized by compounds such as isoamyl acetate [C4], 1-decanol [C50], citronellol [C51], and 1,3-di-tert-butylbenzene [C19]. According to hierarchical clustering analysis (HCA), fermented samples of GTW and WYW were divided into four distinct clusters: cluster I (GTW_D1, D2, D3, D5, D7, D10, and WYW_D1, D2), cluster II (WYW_D20, D30, and D45), cluster III (GTW_D20, D30, and D45), and cluster IV (WYW_D3, D5, D7, and D10), indicating that there was a significant difference in the composition of VFCs produced during GTW and WYW brewing (Figure 5D).

### 3.6. The Characteristic Aroma-Active Compounds between GTW and WYW

To further reveal the characteristic VFCs of GTW and WYW, their intergroup differences in the abundance of volatile compounds were analyzed at day 45 of fermentation using fold change and significance testing (Figure 5E). The significant differential VFCs between WYW and GTW were identified with a fold change threshold of two. The results indicated a markedly higher abundance of most volatile flavor compounds in WYW compared to GTW. Specifically, esters such as ethyl acetate [C1], ethyl butyrate [C2], ethyl octanoate [C20], ethyl caprate [C38], diethyl succinate [C42], ethyl glutarate [C52], ethyl 2-phenylacetate [C53], butyl ethyl succinate [C54], ethyl laurate [C57], and ethyl stearate [C87] were significantly enriched in WYW. Among the alcohols, notable enrichment in WYW encompassed isobutanol [C3], 1-octen-3-ol [C21], [2E]-2-octen-1-ol [C36], and 2,4,7,9-tetramethyl-5-decyn-4,7-diol [C73]. Conversely, within the GTW variety, significant enrichment of volatile flavor compounds was observed, including isoamyl acetate [C4], ethyl heptanoate [C13], ethyl nonanoate [C28], 1-decanol [C50], citronellol [C51], phenethyl acetate [C56], and hexanoic acid [C60]. These findings contribute to a more comprehensive understanding of the nuanced volatile flavor composition differences between GTW and WYW. On the contrary, significant enrichment of some volatile flavor compounds was observed in GTW, including isoamyl acetate [C4], ethyl heptanoate [C13], ethyl nonanoate [C28], 1-decanol [C50], citronellol [C51], phenethyl acetate [C56], and hexanoic acid [C60].

While a variety of differentially volatile compounds were identified in both GTW and WYW, it is crucial to recognize that not all these compounds significantly influence the overall aromatic profile of HQW. In view of the large number of volatile substances, it is convenient to investigate the difference in contribution degree of different compounds to flavor. Odor activity values (OAVs) were computed using the average analytical concentrations in conjunction with established odor thresholds from literature sources. In the context of evaluating the aroma of HQW, it is generally accepted that only those differential VFCs with OAVs exceeding 1 have a notable influence on the aroma composition [34]. 14, and 19 characteristic volatile compounds (OAVs > 1) were identified in GTW and WYW, respectively (Supplementary Table S1). These VFCs may contribute significantly to the flavor of HQW and may explain to some extent the sensory differences between GTW and WYW. The characteristic volatile compounds (OAVs > 1) found in GTW mainly encompassed isoamyl acetate (OAV 6320.95), ethyl caprate (OAV 1053.12), ethyl butyrate (OAV 263.89), ethyl heptanoate (OAV 228.56), and ethyl octanoate (OAV 158.36). In WYW, we found characteristic volatile flavor components such as ethyl caprate (OAV 3697.14), isoamyl acetate (OAV 2688.10), ethyl butyrate (OAV 786.22), ethyl octanoate (OAV 504.98), ethyl heptanoate (OAV 84.27), 2-methoxy-4-vinylphenol (OAV 71.42), and 1-octen-3-ol (OAV 68.93). On the other hand, from the perspective of aroma components and sensory characteristics, the contents of isoamyl acetate, ethyl caprate, ethyl butyrate, ethyl octanoate, and ethyl acetate were much higher than the sensory flavor threshold, and they were the most prominent core VFCs in the two HQWs. Among them, isoamyl acetate and ethyl caprate, as a whole, show a pleasant fruity and floral aroma [35]. In addition, ethyl butyrate presents fruity, citrus, and other fresh flavor [36]. Ethyl octanoate has berry and floral flavors, which plays a key role in improving the sensory quality of wine [35]. As can be seen from Supplementary Table S1, there are significant differences in key VFCs of the two HQWs. Therefore, the selection of different *Jiuqu* for brewing has important theoretical guiding significance for the control of HQW flavor quality. But at the same time, it should be recognized that analysis techniques of VFCs have their limitations, for example, some compounds may be difficult to be collected or are easily decomposed in the analysis process. So, future research work should enrich the research methods of flavoromics and sensoryomics, that is, by combining flavor detection data with flavor sensory data, so as to continuously explore the compounds in the wine body that contribute to flavor perception.

### 3.7. The Dynamics of Microbial Communities during GTW and WYW Brewing

**Figure 6:**
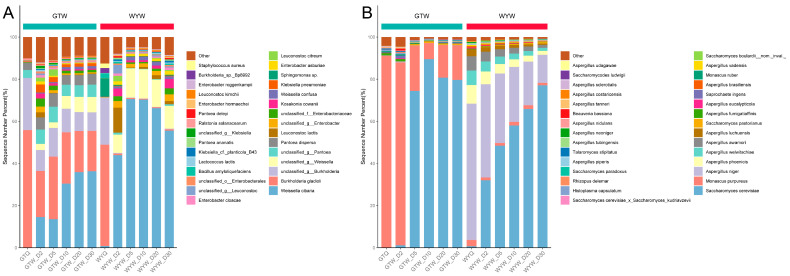
Stacked histogram of the relative abundance of the predominant bacterial (**A**) and fungal (**B**) species during the brewing of GTW and WYW.

The metagomic sequencing of microorganisms involved in brewing has become a bridge between brewing complex microorganisms and flavor components, which helps to understand and reveal the reasons for the differences in VFCs between the Hongqu wines. Therefore, this study analyzed the dynamics of the dominant microbial communities during the brewing process of GTW and WYW at the species level. As depicted in Figure 6A, *Burkholderia gladioli*, *Burkholderia* sp., and *Pantoea* sp. were the predominant bacterial species in GTQ, while in WYQ the predominant bacterial species included *Burkholderia gladioli*, *Burkholderia* sp., and *Sphingomonas* sp. During GTW brewing, *Weissella cibaria* and *Burkholderia gladioli*, etc., were the most predominant bacterial species. As for WYW brewing, the predominant bacterial species included *Weissella cibaria*, *Kosakonia cowanii*, *Enterobacter* sp., *Enterobacter asburiae*, *Leuconostoc lactis*, etc. Figure 6B shows the dynamic changes in dominant fungal community at a species level throughout the GTW and WYW brewing processes. The predominant fungal species in the GTW brewing process included *Monascus purpureus* and *Saccharomyces cerevisiae*. In addition to *Saccharomyces cerevisiae* and *Monascus purpureus*, *Aspergillus niger*, *Aspergillus welwitschiae*, *Aspergillus awamori*, etc., were identified as the predominant fungal species in WYW brewing, but they were difficult to detect in the GTW brewing process.

**Figure 7:**
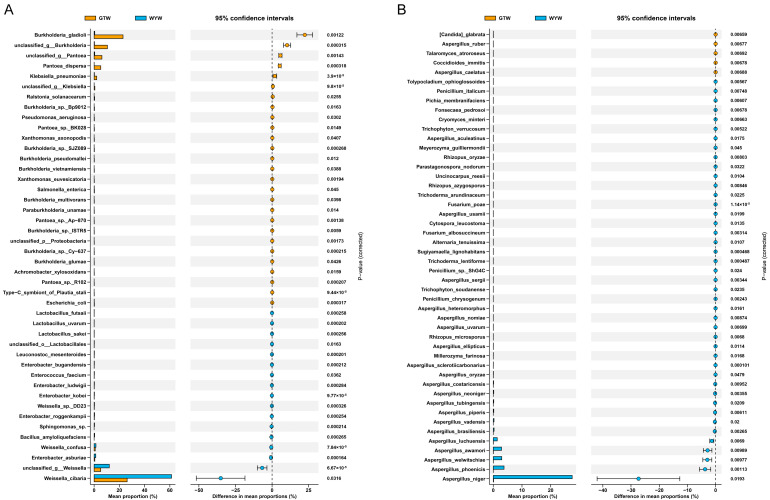
Visualization of the differences in the relative abundance of bacterial (**A**) and fungal (**B**) species between GTW and WYW.

To further elucidate the differences in microbial composition in the brewing of GTW and WYW, STAMP software was used to conduct differential analysis and visualization of microbial species in the brewing of GTW and WYW. As illustrated in Figure 7, during the brewing process of GTW and WYW, there were significant differences in the abundance and distribution of microbiomes, mainly reflected in 45 bacterial species and 48 fungal species. To be specific, *Burkholderia gladioli*, *Pantoea dispersa*, *Klebsiella pneumoniae*, *Burkholderia* sp. SJZ089, and *Xanthomonas euvesicatoria*, etc., were more abundant in GTW brewing, while *Lactobacillus futsaii*, *Leuconostoc mesenteroides*, *Weissella confusa*, *Enterobacter ludwigii*, *Sphingomonas* sp., and *Bacillus amyloliquefaciens*, etc., were significantly enriched in WYW brewing (Figure 7A). It is obvious that the abundance in the majority of fungal species in WYW brewing were notably higher than those in GTW brewing. Fungal species such as *[Candida] glabrata*, *Aspergillus ruber*, *Talaromyces atroroseus*, *Coccidioides immitis*, and *Aspergillus caelatus* were significantly enriched in GTW brewing, while *Fusarium poae*, *Aspergillus sclerotiicarbonarius*, *Cytospora leucostoma*, *Alternaria tenuissima*, *Trichoderma lentiforme*, and *Penicillium chrysogenum*, etc., were significantly enriched in WYW brewing (Figure 7B).

### 3.8. Functional Genes Related to Biogenic Amines Metabolism

BAs may be present in the raw materials for brewing but are formed mainly during fermentation by decarboxylation of precursor amino acids [30]. Rice wine brewing is carried out under natural open conditions with the synergistic participation of multiple microorganisms and enzymes; the microorganisms produce amino acid decarboxylase, which acts on amino acids in the fermentation broth during the fermentation process, catalyzing the decarboxylation of amino acids to produce BAs [31]. The degradation of BAs in fermented foods mainly depends on the amine oxidase produced by the fermentation microbial flora [37]. For example, monoamine oxidase (MAO) and diamine oxidase (DAO) can degrade histamine and other BAs [38]. Therefore, to further understand the formation and degradation mechanisms of BAs during GTW and WYW brewing, the functional genes related to the metabolism of amino acids and BAs were annotated to KEGG orthologues database.

**Figure 8:**
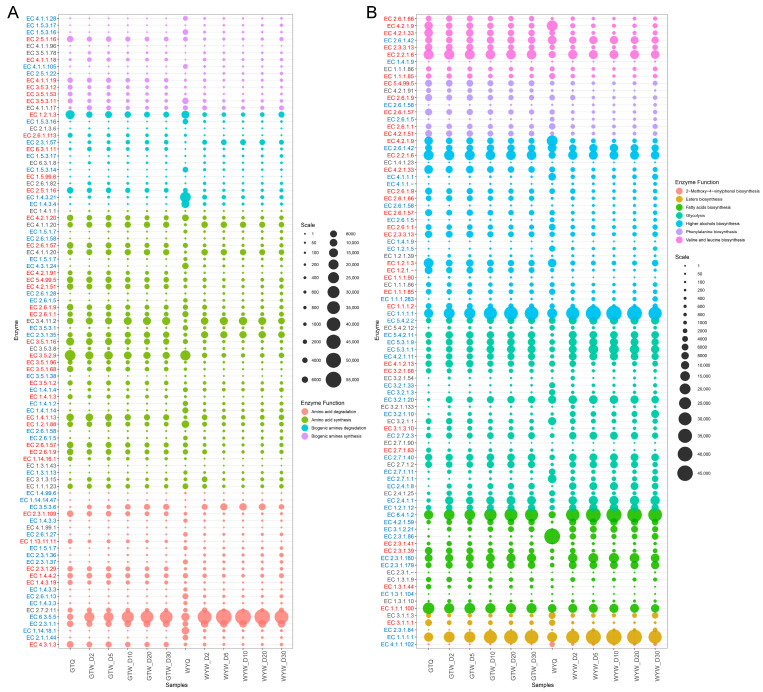
Bubble chart of the abundance of microbial genes for enzymes closely related to the metabolism of amino acids and biogenic amines (**A**), and the metabolism of characteristic volatile components (**B**) during the brewing of GTW and WYW.

Microbial annotation was performed for the functional genes for the metabolism of amino acids and BAs (as shown in Supplementary Table S2). The abundances of coding genes corresponding to enzymes involved in the metabolic pathway of amino acids and BAs were displayed in Figure 8A. It is clear that there were striking differences in the abundance of functional genes between GTW and WYW brewing. Putrescine and cadaverine are the two most abundant BAs in HQW. Among them, putrescine is converted by spermidine. Specifically, spermidine decarboxylase [EC 4.1.1.19] acts on spermidine to generate agmatine, which is then deaminated by agmatinase [EC 3.5.3.11] and agmatine deiminase [EC 3.5.3.12], and finally synthesized by n-carbamoyl-putrescine aminoase [EC 3.5.1.53] [39,40]. As shown in Figure 8A, the abundance of genes encoding the four enzymes mentioned above was significantly higher in GTW brewing than in WYW, which resulted in significantly higher levels of putrescine in GTW than in WYW. In addition, microbial annotation found that *Pantoea dispersa*, *Klebsiella pneumoniae*, *Burkholderia gladioli*, *Enterobacter chengduensis*, *Kosakonia cowanii*, *Enterobacter hormaechei*, *Aspergillus phoenicis*, *Lactococcus lactis*, etc., were the main microorganisms encoding the above four enzyme genes (Supplementary Table S2). Cadaverine is mainly produced by direct decarboxylation of L-lysine by lysine decarboxylase [EC 4.1.1.18] [41]. The microorganisms that are closely related to the gene encoding EC 4.1.1.18 are mainly *Pantoea dispersa*, *Klebsiella pneumoniae*, *Kosakonia cowani*, *Pantoea deleyi*, *Cronobacter malonaticus*, etc. (Supplementary Table S2). In addition, it can be seen from Figure 6 that microorganisms closely related to BAs production, such as *Pantoea dispersa*, *Lactococcus lactis*, etc., showed a gradual increase throughout the brewing process. No functional genes associated with the direct synthesis of tyramine were detected in this study, but primary amine oxidase [EC 1.4.3.21] and monoamine oxidase [EC 1.4.3.4] associated with tyramine degradation were detected. The results showed that the abundance of genes encoding the above two enzymes were significantly higher in WYW than in GTW, which was conducive to tyramine degradation in WYW. In summary, the results of this study help to reveal the metabolic mechanism of BA synthesis by functional microbial fermentation, provide a scientific basis for the establishment of BA reduction regulation strategies, and lay a theoretical basis for the subsequent isolation and screening of functional strains, so as to achieve the control of BA metabolic pathways during HQW brewing.

### 3.9. Functional Genes Related to Flavor Components Metabolism

The enzymes responsible for characteristic flavor metabolic pathways were systematically identified using the KEGG database and relevant academic literature. Therefore, we used a bubble chart to show the types and abundance of major enzymes in the pathway (Figure 8B). Microbial annotation was conducted on functional genes involved in flavor component metabolism (Supplementary Table S3). Glutinous rice is rich in starch, and it is converted to dextrin and maltose under the enzymatic action of α-amylase [EC 3.2.1.1] or α-glucan 1,4-α-maltose hydrolase [EC 3.2.1.133]. Subsequently, the synergistic action of starch α-1,6-glucosidase [EC 3.2.1.33] and α-1,4-glucosidase [EC 3.2.1.3] results in the hydrolysis of the α-1,6-glucosidic bond to produce glucose. Comparative analysis of gene abundance showed that the abundance of enzymes related to starch metabolism in GTW was generally lower than that in WYW, especially the key enzymes mediating the conversion of starch to glucose, such as EC 3.2.1.133, EC 3.2.1.33 and EC 3.2.1.3. The results of the functional gene abundance showed that the microbial community in WYW brewing used starch more efficiently. Microbial annotation of functional genes found that EC 3.2.1.133 in WYW brewing was predominantly annotated by *Leuconostoc lactis* and *Lactococcus garvieae*, while EC 3.2.1.33 was mainly derived from *Monascus purpureus* and *Saccharomyces cerevisiae* in WYW brewing (Supplementary Table S3). Additionally, key enzymes in the glycolysis pathway, i.e., glycerol-aldehyde-3-phosphate dehydrogenase (phosphorylating) [EC 1.2.1.12], fructose-6-phosphate kinase [EC 2.7.1.11], pyruvate kinase [EC 2.7.1.40], and phosphoenolpyruvate carboxykinase [EC 4.2.1.11], showed higher abundance in WYW brewing (Figure 8B). EC 2.7.1.11 was primarily annotated by *Saccharomyces cerevisiae*, unclassified_f_*Enterobacteriaceae*, *Aspergillus niger*, *Enterobacter*_sp._UCD-UG_FMILLET, etc. EC 2.7.1.40 was mainly associated with *Weissella cibaria*, *Saccharomyces cerevisiae*, *Leuconostoc* sp., *Lichtheimia ramosa*, *Enterobacter* sp., and *Burkholderia gladioli*, and were closely related (Supplementary Table S3). The results of this study also further explained that the consumption rate of reducing sugar in WYW brewing is greater than that of GTW (Figure 1B).

HAs are considered to be the main components essential to the characteristic VFCs of HQW, which are mainly formed through the anabolic pathway (Harris pathway) and degradation metabolic pathway (Ehrlich pathway) [16]. Of these, 75% of HAs are derived from the Harris pathway and 25% from the Ehrlich pathway [42], and this paper is mainly discussing the anabolic pathway. The anabolic pathway mainly involves the production of pyruvic acid through glycolysis, which is then converted to the corresponding α-keto acid, and further decarboxylated to form aldehydes and then reduced to the corresponding alcohols [6]. Therefore, the efficient expression of enzymes related to glycolysis pathway in the early stage of rice wine brewing contributes to the high production of HAs by *Saccharomyces cerevisiae*. Enzymes involved in the conversion of α-keto acids to HAs mainly include pyruvate decarboxylase [EC 4.1.1.1, catalyzing α-keto acids to corresponding aldehydes] and alcohol dehydrogenase [EC 1.1.1.1, catalyzing isobutyraldehyde, isoamylaldehyde and phenylacetaldehyde to isobutanol, isoamylalcohol and phenylethanol, respectively]. EC 4.1.1.1 was mainly annotated by *Saccharomyces cerevisiae*, *Aspergillus piperis*, *Penicillium coprophilum*, and *Rhizopus delemar*. EC 1.1.1.1 was mainly produced by microorganisms such as *Weissella cibaria*, *Weissella*_sp._DD23, *Achromobacter xylosoxidans*, *Klebsiella pneumoniae*, and *Leuconostoc citreum* (Supplementary Table S3). As can be seen from Figure 8B, the abundance of coding genes corresponding to EC 4.1.1.1 and EC 1.1.1.1 in GTW brewing was remarkably lower than that of WYW, resulting in a significant difference in HA content between the two rice wines. Among them, the highest content of 3-methyl-1-butanol was found in GTW and WYW, which accounted for more than 50% of the total HAs, and the abundance of genes related to the biosynthesis of 3-methyl-1-butanol was found to be enormously different between the two types of HQW. The synthesis of 3-methyl-1-butanol involves the conversion of leucine to α-ketoisocaproate, catalyzed by branched-chain amino acid transaminase [EC 2.6.1.42] or leucine dehydrogenase [EC 1.4.1.9]. Subsequently, α-ketoisocaproate is transformed into isovaleraldehyde by EC 4.1.1.1 and further reduced to 3-methyl-1-butanol by EC 1.1.1.1, alcohol dehydrogenase (NADP+) [EC 1.1.1.2], or methyl ethyl ketone reductase (NADPH) [EC 1.1.1.283] [43]. The relevant enzymes are notably enriched in WYW (Figure 8B) and annotated by diverse species (Supplementary Table S3), contributing significantly to the heightened accumulation of 3-methyl-1-butanol in WYW during fermentation. Noteworthy is the conspicuous difference in EC 1.4.1.9, predominantly annotated by *Bacillus amyloliquefaciens* and *Rhodococcus* sp. (Supplementary Table S3). Moreover, throughout the fermentation process of WYW, brewing yeast assumes a pivotal role in the complete conversion of leucine to 3-methyl-1-butanol, underscoring its importance in the formation of this compound [16].

Esters are essential VFCs in rice wine, which have fruity, sweet, and floral flavor, enhancing the aroma of alcoholic beverages [44]. There are two primary pathways of ester formation: esterification between alcohols and acids mediated by carboxylesterase [EC 3.1.1.1], and condensation between acetyl-CoA and alcohols catalyzed by alcohol O-acetyltransferase [EC 2.3.1.84] [45]. Notably, the abundance of genes encoding EC 3.1.1.1 and EC 2.3.1.84 showed significant differences between GTW and WYW. Saccharomyces cerevisiae in HQW brewing is an important ester producing yeast, and the EC 2.3.1.84 encoded by this type of yeast is conducive to the synthesis of acetic esters such as ethyl acetate and isoamyl acetate [46]. Interestingly, the gene encoding EC 2.3.1.84 was significantly more enriched in WYW than in GTW, resulting in more ester production in WYW brewing, which was consistent with the findings in Figure 4. Conversely, the decomposition and transformation of esters in HQW may also occur, which is closely related to the hydrolysis of esters into alcohols and carboxylic acids catalyzed by EC 3.1.1.1 [47], which were mainly encoded by *Burkholderia* sp., *Burkholderia gladioli*, *Aspergillus niger*, unclassified_g_*Bacillus*_f_*Bacillaceae*, and *Bacillus ginsengihumi* (Supplementary Table S3). EC 3.1.1.1 is considerably enriched in GYW. However, being a bidirectional enzyme, it is challenging to directly infer the quantity of lipids produced. Additionally, the study also identified a gene encoding triacylglycerol lipase [EC 3.1.1.3], whose main function is to hydrolyze triacylglycerol into glycerol and free fatty acids, with both hydrolysis and transacylation activities [48]. In this study, EC 3.1.1.3 was mainly produced by microorganisms such as *Burkholderia gladioli*, unclassified_o_*Enterobacterales*, *Enterobacter cloacae*, *Aspergillus phoenicis*, etc. (Supplementary Table S3). Given that organic acids are key precursors for ester formation, we also explored functional genes corresponding to enzymes associated with fatty acid synthesis. Fatty acid synthase [EC 2.3.1.86] can catalyze the production of long chain fatty acids such as palmitic acid and stearic acid, providing a more harmonious flavor for HQW. In the brewing process of HQW, functional microorganisms with the potential to produce EC 2.3.1.86 mainly include *Aspergillus niger*, *Lichtheimia ramosa*, *Saccharomyces cerevisiae*, *Aspergillus welwitschiae*, *Rhizopus delemar*, and *Monascus purpureus* (Supplementary Table S3). Acetyl-CoA carboxylase [EC 6.4.1.2], 12-oxophytodienoate reductase [EC 3.1.2.21], and acyl-[acyl-carrier-protein] hydrolase [EC 3.1.2.14] participate in the synthesis of fatty acids. Microbial annotation results showed that they were mainly associated with Weissella sp. DD23, *Weissella* sp., *Saccharomyces cerevisiae*, *Leuconostoc lactis*, *Lichtheimia ramosa*, and *Leuconostoc* sp., which are closely related (Supplementary Table S3).

In this study, GC-MS analysis of volatile substances showed that 2-methoxy-4-vinylphenol [C80] was significantly enriched in WYW compared to GTW. A previous study indicated that ferulic acid decarboxylase [EC 4.1.1.102] facilitates the conversion of phenolic compounds such as 4-coumaric acid and ferulic acid to 4-vinylphenol and 2-methoxy-4-vinylphenol [49]. As can be seen from Figure 8B, the abundance of coding gene corresponding to EC 4.1.1.102 in WYW is significantly higher than that in GTW, which aptly explains why the content of 2-methoxy-4-vinylphenol [C80] was relatively high in WYW. As shown in Supplementary Table S3, EC 4.1.1.102 was mainly produced by *Monascus purpureus* and *Saccharomyces cerevisiae* in WYW brewing. In summary, these findings can partially explain the metabolic pathways responsible for synthesis of HAs, esters, and other flavor compounds, as well as the related microbial regulatory mechanisms during the brewing process of HQW, and provide an important scientific basis for the subsequent targeted regulation and enhancement of flavor quality.

## 4. Conclusions

This study compared the differences in physicochemical properties, AAs, BAs, HAs, VFCs, and microbial communities in GTW and WYW brewing, which is helpful to understand the influence of microbial communities on the flavor quality of HQW, and provide an important scientific basis for the upgrading of the HQW industry. Distinct differences were observed in the volatile flavor profiles of GTW and WYW, wherein volatile compounds, such as ethyl acetate, 2-nonanone, and 2-ethylhexanol, exhibited heightened concentrations in WYW. In contrast, characteristic ester flavor components, including ethyl caproate, ethyl heptanoate, and acetic acid demonstrated significantly higher yields in GTW. The metagenomic analysis, based on high-throughput sequencing, revealed substantial disparities in microbial composition characteristics between the traditional brewing processes of GTW and WYW. Furthermore, bioinformatics analyses based on the NR database and KEGG database elucidated the microbial enzymes involved in the metabolism of amino acids, BAs, Has, and volatile flavor compounds during HQW brewing. This study furnishes critical scientific insights for enhancing the flavor quality of HQW, thereby establishing a robust foundation for the sustainable development of the rice wine industry. However, metagenomic sequencing also presents associated challenges, such as the complexity of sample preparation, the bias of the DNA extraction process, and the reliance on existing databases for data interpretation. Therefore, multi-dimensional methods such as metagenomics, metaproteomics, and DNA-based stable isotope probing should be used in future studies to deepen the comprehensive understanding of microbial functions and influencing mechanisms in rice wine.

## Data Availability

The original contributions presented in the study are included in the article/Supplementary Materials, further inquiries can be directed to the corresponding author.

## Supplementary Materials

The following supporting information can be downloaded at: https://www.mdpi.com/article/10.3390/foods13193114/s1, Table S1: Characteristic volatile flavor components (VFCs) in GTW and WYW screened by SPME-GC/MS assay and OAV calculation; Table S2: Microbial contributions to amino acids and biogenic amines metabolizing enzymes in GTW and WYW brewing; Table S3: Microbial contributions to flavor metabolizing enzymes in GTW and WYW brewing.
