# Supplementary material for: Metagenomic and Metabolomic Profiling Reveals the Differences of Flavor Quality between Hongqu Rice Wines Fermented with Gutian Qu and Wuyi Qu
Source: Foods. 2024 Sep 29;13(19):3114. doi: 10.3390/foods13193114 (PMC11475954; doi:10.3390/foods13193114)
Supplement: Supplementary file 1 [file foods-13-03114-s001.zip › foods-3229310-supplementary.pdf]

Supplementary materials

Table S1. Characteristic volatile flavor components (VFCs) in GTW and WYW screened by SPME-GC/MS assay and OAV calculation.

No.	Compounds	CAS	RI	Concentration (ug/L)		Threshold	OAV	
				WYW_D45	GTW_D45	(mg/L)#	WYW_D45	GTW_D45
**Acid**								
C37	γ-Hydroxybutyric acid	591-81-1	1623	14.21±1.73	6.78±0.5	-	-	-
C72	Octanoic acid	124-07-2	2065	1091±26.74	349.39±51.22	3	0.364	0.116
C82	n-Decanoic acid	334-48-5	2280	59785.04±146.6	11408.41±4078.87	10	5.979	1.141
C89	Dodecanoic acid	143-07-7	2495	28.34±1.81	13.67±5.51	10	0.003	0.001
**Alcohol**								
C3	Isobutanol	78-83-1	1095	64002.17±2374.71	21780.91±16540.67	6.5052	9.839	3.348
C7	1-Pentanol	71-41-0	1250	n.d.	258.04±51.16	0.1502	-	1.718
C21	1-Octen-3-ol	3391-86-4	1448	103.39±7.16	36.38±3.1	0.0015	68.927	24.255
C36	(2E)-2-Octen-1-ol	18409-17-1	1607	29.07±0.61	n.d.	0.04	0.727	-
C43	(3Z)-3-Nonen-1-ol	10340-23-5	1672	n.d.	3.08±0.32	-	-	-
C46	3-(Methylsulfanyl)-1-propanol	505-10-2	1709	2348.78±156.81	1152.95±171.92	0.12323	19.060	9.356
C50	1-Decanol	112-30-1	1751	n.d.	8.67±1.05	0.775	-	0.011
C51	Citronellol	106-22-9	1763	0.73±0.06	2.15±0.36	0.062	0.012	0.035
C58	Geraniol	106-24-1	1838	6.66±0.55	n.d.	0.0066	1.009	-
C73	2,4,7,9-Tetramethyl-5-decyn-4,7-diol	126-86-3	1976	96.69±13.45	3.56±0.57	-	-	-
C85	Farnesol	4602-84-0	2349	8.89±0.73	n.d.	1	0.009	-

Ester

C1	Ethyl acetate	141-78-6	906	196740.94±34968.17	88800.06±8597.6	7.5	26.232	11.840
C2	Ethyl butyrate	105-54-4	1039	707.6±354.38	237.5±175.61	0.0009	786.220	263.890
C4	Isoamyl acetate	123-92-2	1125	403.22±17.44	948.14±92.06	0.00015	2688.103	6320.952
C13	Ethyl heptanoate	106-30-9	1330	160.11±28.05	434.26±52.92	0.0019	84.266	228.556
C20	Ethyl octanoate	106-32-1	1429	9746.12±201.35	3056.34±235.04	0.0193	504.980	158.360
C28	Ethyl nonanoate	123-29-5	1528	n.d.	99.9±7.76	0.377	-	0.265
C38	Ethyl caprate	110-38-3	1632	18485.69±3276.89	5265.61±1139.65	0.005	3697.138	1053.122
C42	Diethyl succinate	123-25-1	1674	21411.91±922.29	1525.91±46.2	100	0.214	0.015
C52	Ethyl glutarate	818-38-2	1777	10.85±0.63	4.75±1.48	-	-	-
C53	Ethyl 2-phenylacetate	101-97-3	1780	875.43±42.22	139.08±5.58	0.15555	5.628	0.894
C54	Butyl ethyl succinate	67233-92-5	1759	139.68±8.01	16.08±1.37	-	-	-
C56	Phenethyl acetate	103-45-7	1808	231.64±9.3	560.37±22.91	0.24959	0.928	2.245
C57	Ethyl laurate	106-33-2	1837	47.78±4.34	13.21±2.66	5.9	0.008	0.002
C61	n-Butyl butanoate	109-21-7	1863	462.19±32.98	33.95±1.55	0.4	1.155	0.085
C62	Cyclopentyl butyrate	6290-13-7	1876	14.34±0.64	1.99±0.34	-	-	-
C63	Ethyl 3-phenylpropanoate	2021-28-5	1879	1.94±0.38	n.d.	0.0016	1.212	-
C65	Ethyl 3-methylbutyl succinate	28024-16-0	1897	400.52±19.41	29.84±4.03	-	-	-
C71	Ethyl myristate	124-06-1	2043	24.88±3.9	3.55±1.17	4	0.006	0.001
C74	Ethyl 3-hydroxytridecanoate	107141-15-1	1886	116.64±5.01	34.1±9.96	-	-	-
C81	Ethyl palmitate	628-97-7	2252	18291.63±3034.05	5502.63±3114.87	2	9.146	2.751
C84	Ethyl 9-hexadecenoate	54546-22-4	2327	22.8±3.6	n.d.	10	0.002	-
C87	Ethyl stearate	111-61-5	2462	132.84±22.96	29.85±6.88	0.51	0.260	0.059

C90	Ethyl linoleate	544-35-4	2533	13553.57±4656.02	n.d.	0.45	30.119	-
**Ketone**								
C8	3-Octanone	106-68-3	1255	23.24±15.27	n.d.	0.0214	1.086	-
C14	6-Methyl-5-hepten-2-one	110-93-0	1333	12.44±1.21	n.d.	0.068	0.183	-
C17	2-Nonanone	821-55-6	1379	55.38±1.02	5.96±0.24	0.2	0.277	0.030
C40	Acetophenone	98-86-2	1641	18.56±1.33	8.81±1.37	0.245	0.076	0.036
**Phenol**								
C49	4-(Methylsulfanyl)phenol	1073-72-9	1744	17.88±2.79	3.98±0.9	0.8	0.022	0.005
C69	Phenol	108-95-2	2006	n.d.	1.36±0.34	58.58525	-	-
C78	4-Ethylphenol	123-07-9	2180	182.81±8.26	n.d.	0.013	14.062	-
C80	2-Methoxy-4-vinylphenol	7786-61-0	2200	858.41±108.83	12.67±2.21	0.01202	71.415	1.054
**Others**								
C9	2-Methylpyrazine	109-08-0	1263	n.d.	191.91±62.35	30	-	0.006
C19	1,3-Di-tert-butylbenzene	1014-60-4	1419	12.08±5.56	n.d.	-	-	-
C86	2,3-Dihydro-1-benzofuran	496-16-2	2402	20.4±3.24	7.69±3.17	-	-	-

Threshold were from the literatures [Yu et al., 2019; Liu et al., 2022; Moreno et al., 2005; Salo et al., 2006], or from Compilations of Odour Threshold Values in Air, Water and Other media [Gemert, 2003] (Second Enlarged and Revised Edition), ISBN 978-7-03-058320-8

References:

Yu, H., Xie, T., Xie, J., Ai, L., Tian, H., Characterization of key aroma compounds in Chinese rice wine using gas chromatography-mass spectrometry and gas chromatography-olfactometry[J]. Food Chemistry, 2019, 293: 8-14.

Liu, Z.; Chen, F.; Sun, J.; Ni, L., Dynamic changes of volatile and phenolic components during the whole manufacturing process of Wuyi Rock tea (Rougui)[J]. Food Chemistry, 2022, 367.

Moreno, J. A.; Zea, L.; Moyano, L.; Medina, M., Aroma compounds as markers of the changes in sherry wines subjected to biological ageing[J]. Food Control, 2005, 16 (4), 333-338.

Salo, P.; NykÄNen, L.; Suomalainen, H. Odor thresholds and relative intensities of volatile aroma components in an artificial beverage imitating whisky[J]. Journal of Food Science 2006, 37, 394-398.

Gemert, L.J.V. Compilations of odour threshold values in air, water and other media. 2003.

Table S2. Microbial contributions to amino acids and biogenic amines metabolizing enzymes in GTW and WYW brewing.

Enzyme function	Enzyme name	Enzyme number	GTW	WYW
			Distribution of microbes (>1%)	Distribution of microbes (>1%)
Biogenic amines synthesis	aromatic-L-amino-acid decarboxylase	4.1.1.28	*Monascus purpureus (100%)*	*Aspergillus niger (96.09%), Monascus purpureus (3.91%)*
	ornithine decarboxylase	4.1.1.17	*Pantoea dispersa (41.48%), Kosakonia cowanii (9.58%), Klebsiella pneumoniae (9.55%), Enterobacter mori (7.50%), Enterobacter hormaechei (6.66%), Enterobacter roggenkampii (5.95%), Enterobacter cloacae (4.37%), Cronobacter malonaticus (2.85%), Enterobacter bugandensis (2.08%), Franconibacter pulveris (1.86%), unclassified_f_Enterobacteriaceae (1.78%), Enterobacter_sp._SES19 (1.36%)*	*Aspergillus niger (27.61%), Saccharomyces cerevisiae (16.65%), Enterobacter cloacae (14.08%), Enterobacter mori (10.86%), Kosakonia cowanii (10.80%), Enterobacter asburiae (4.70%), Enterobacter bugandensis (2.72%), Enterobacter hormaechei (2.58%), Pantoea deleyi (1.98%), Monascus purpureus (1.83%), Enterobacter_sp._18A13 (1.74%), Pantoea ananatis (1.00%)*
	agmatinase	3.5.3.11	*Burkholderia gladioli (81.88%), unclassified_c_Gammaproteobacteria (6.45%), Enterobacter chengduensis (3.74%), Leclercia adecarboxylata (3.64%), unclassified_g_Pantoea (1.98%)*	*Aspergillus niger (31.82%), Aspergillus phoenicis (31.24%), Enterobacter chengduensis (7.80%), Escherichia coli (6.12%), unclassified_c_Gammaproteobacteria (5.43%), Burkholderia gladioli (4.82%), Aspergillus udagawae (2.95%), Monascus purpureus (2.12%), Aspergillus awamori (2.06%), unclassified_g_Pantoea (1.90%), Monascus ruber (1.51%), Bacillus amyloliquefaciens (1.50%)*
	N-carbamoylputrescine amidase	3.5.1.53	*Burkholderia gladioli (90.93%), Klebsiella_cf._planticola_B43 (9.03%)*	*unclassified_f_Enterobacteriaceae (78.93%), Burkholderia gladioli (20.40%)*

	non-specific polyamine oxidase	1.5.3.17	*Saccharomyces cerevisiae (100%)*	*Saccharomyces cerevisiae (99.16%)*
	agmatine deiminase	3.5.3.12	*Burkholderia gladioli (92.43%), Kosakonia cowanii (4.85%), Lactococcus lactis (2.37%)*	*Kosakonia cowanii (54.10%), Burkholderia gladioli (30.79%), Lactococcus lactis (9.97%), Burkholderia cepacia (3.97%)*
	arginine decarboxylase	4.1.1.19	*unclassified_g_Burkholderia (59.21%), Pantoea dispersa (24.00%), Klebsiella pneumoniae (8.53%), unclassified_g_Enterobacter (2.71%), Pantoea ananatis (1.38%), Cronobacter sakazakii (1.06%)*	*unclassified_g_Enterobacter (22.48%), unclassified_g_Burkholderia (16.63%), Klebsiella pneumoniae (13.03%), Pantoea deleyi (11.64%), Enterobacter hormaechei (7.40%), unclassified_f_Enterobacteriaceae (5.23%), Mixta calida (4.56%), Bacillus amyloliquefaciens (4.36%), Pantoea dispersa (2.12%), Cronobacter sakazakii (2.11%), Pantoea ananatis (2.09%), Kosakonia sacchari (1.91%), Bacillus ginsengihumi (1.31%), Variovorax_sp._SCN_67-85 (1.12%)*
	spermine synthase	2.5.1.22	*Saccharomyces cerevisiae (100.00%)*	*Saccharomyces cerevisiae (100.00%)*
	L-tryptophan decarboxylase	4.1.1.105	*Monascus purpureus (100.00%)*	*Aspergillus niger (96.09%), Monascus purpureus (3.91%)*
	lysine decarboxylase	4.1.1.18	*Pantoea dispersa (49.69%), Klebsiella pneumoniae (22.86%), Enterobacter cloacae (14.47%), Kosakonia cowanii (4.57%), Cronobacter malonaticus (1.82%), unclassified_g_Kosakonia (1.73%), Pantoea deleyi (1.65%)*	*Enterobacter cloacae (55.40%), Kosakonia cowanii (15.50%), Pantoea deleyi (6.99%), unclassified_f_Enterobacteriaceae (6.51%), Klebsiella pneumoniae (5.79%), Pantoea ananatis (2.78%), Enterobacter kobei (2.73%), unclassified_g_Pantoea (2.69%), Enterobacter huaxiensis (1.00%)*
	spermidine synthase	2.5.1.16	*unclassified_g_Burkholderia (39.27%), Burkholderia gladioli (35.08%), Pantoea_sp._ICBG_985 (18.75%),*	*Lichtheimia ramosa (40.10%), Saccharomyces cerevisiae (28.91%), Kosakonia cowanii (9.63%), Burkholderia gladioli (6.16%), unclassified_f_Enterobacteriaceae (4.22%),*

			unclassified_f_Enterobacteriaceae (3.09%), Kosakonia cowanii (1.46%)	*Enterobacter hormaechei (2.94%), unclassified_g_Bacillus_f_Bacillaceae (1.96%), Burkholderia pyrrocinia (1.20%), Burkholderia_sp._Bp8992 (1.10%)*
	spermine oxidase	1.5.3.16	-	*Aspergillus niger (52.16%), Aspergillus welwitschiae (47.84%)*
	glutathionyl spermidine amidase	3.5.1.78	*Enterobacter cloacae (36.07%), unclassified_f_Enterobacteriaceae (32.61%), Enterobacter_sp._WCHEn090032 (13.14%), Klebsiella pneumoniae (8.32%), Enterobacter_sp._50588862 (3.96%), Salmonella enterica (3.53%), [Enterobacter] lignolyticus (1.48%)*	*Enterobacter cloacae (26.26%), Enterobacter_sp._WCHEn090032 (25.66%), unclassified_g_Enterobacter (24.51%), Salmonella enterica (9.95%), Enterobacter_sp._50588862 (7.60%), [Enterobacter] lignolyticus (2.94%), unclassified_f_Enterobacteriaceae (2.29%)*
	carboxynorspermidine decarboxylase	4.1.1.96	*Bacillus ginsengihumi (100.00%)*	*Bacillus ginsengihumi (100.00%)*
	tyrosine decarboxylase	4.1.1.25	-	-
Biogenic amines degradation	monoamine oxidase	1.4.3.4	*Monascus purpureus (96.18%), Aspergillus flavus (3.82%)*	*Aspergillus niger (77.33%), Monascus purpureus (21.30%)*
	primary-amine oxidase	1.4.3.21	*unclassified_g_Klebsiella (33.00%), Enterobacter asburiae (16.48%), Monascus purpureus (13.98%), Enterobacter cloacae (13.72%), Enterobacter ludwigii (11.33%), Enterobacter kobei (6.26%), Enterobacter hormaechei (4.75%)*	*Aspergillus niger (55.08%), Aspergillus awamori (18.03%), Aspergillus welwitschiae (17.23%), Monascus purpureus (3.24%), Enterobacter asburiae (2.00%), Enterobacter kobei (1.28%), Enterobacter ludwigii (1.05%)*
	spermidine synthase	2.5.1.16	*unclassified_g_Burkholderia (39.27%), Burkholderia gladioli (35.08%), Pantoea_sp._ICBG_985 (18.75%), unclassified_f_Enterobacteriaceae (3.09%), Kosakonia cowanii (1.46%)*	*Lichtheimia ramosa (40.10%), Saccharomyces cerevisiae (28.91%), Kosakonia cowanii (9.63%), Burkholderia gladioli (6.16%), unclassified_f_Enterobacteriaceae (4.22%), Enterobacter hormaechei (2.94%), unclassified_g_Bacillus_f_Bacillaceae (1.96%),*

				Burkholderia pyrrocinia (1.20%), Burkholderia_sp._Bp8992 (1.10%)
	putrescine-2-oxoglutarate transaminase	2.6.1.82	*Klebsiella pneumoniae (46.78%), Klebsiella_cf._planticola_B43 (21.76%), Lactococcus lactis (6.77%), Kosakonia cowanii (6.71%), Franconibacter pulveris (4.99%), Cronobacter dublinensis (3.61%), Enterobacter roggenkampii (3.05%), Citrobacter amalonaticus (2.10%), Digitaria exilis (1.97%)*	*Klebsiella_cf._planticola_B43 (43.29%), Kosakonia cowanii (14.29%), Klebsiella pneumoniae (13.81%), Enterobacter roggenkampii (6.41%), Cronobacter dublinensis (6.31%), Digitaria exilis (6.15%), Citrobacter amalonaticus (4.06%), Lactococcus lactis (2.12%), Enterobacter kobei (1.26%)*
	spermidine dehydrogenase	1.5.99.6	*Cronobacter sakazakii (72.89%), Cronobacter dublinensis (27.11%)*	-
	polyamine oxidase (propane-1,3-diamine-forming)	1.5.3.14	-	*Aspergillus niger (52.16%), Aspergillus welwitschiae (47.84%)*
	glutathionylspermidine synthase	6.3.1.8	*unclassified_f_Enterobacteriaceae (32.61%), Enterobacter cloacae (22.99%), unclassified_g_Enterobacter (13.45%), Enterobacter_sp._WCHEn090032 (13.14%), Klebsiella pneumoniae (8.32%), Enterobacter_sp._50588862 (3.96%), Salmonella enterica (3.53%), [Enterobacter] lignolyticus (1.48%)*	*Enterobacter cloacae (26.26%), Enterobacter_sp._WCHEn090032 (25.66%), unclassified_g_Enterobacter (24.51%), Salmonella enterica (9.95%), Enterobacter_sp._50588862 (7.60%), [Enterobacter] lignolyticus (2.94%), unclassified_f_Enterobacteriaceae (2.29%)*
	non-specific polyamine oxidase	1.5.3.17	*Saccharomyces cerevisiae (100.00%)*	*Saccharomyces cerevisiae (99.16%)*
	glutamate-putrescine ligase	6.3.1.11	*Pantoea dispersa (42.27%), Leclercia adecarboxylata (30.48%), unclassified_f_Enterobacteriaceae (9.45%), Cronobacter malonaticus (4.05%), Enterobacter cloacae (3.71%), Enterobacter*	*unclassified_g_Enterobacter (32.72%), unclassified_o_Enterobacterales (31.49%), Enterobacter roggenkampii (9.21%), Enterobacter_sp._M4-VN (8.59%), Pantoea deleyi (3.89%), Pantoea ananatis (3.70%),*

			asburiae (2.97%), unclassified_g_Enterobacter (2.71%), Enterobacter roggenkampii (1.15%), Enterobacter kobei (1.04%), Franconibacter pulveris (1.01%)	*Enterobacter cloacae (2.96%), unclassified_f_Enterobacteriaceae (2.71%), Enterobacter kobei (2.34%), unclassified_g_Pantoea (1.86%)*
	diamine N-acetyltransferase	2.3.1.57	*Weissella cibaria (81.36%), Leuconostoc citreum (4.26%), unclassified_g_Enterobacter (3.69%), unclassified_f_Enterobacteriaceae (3.46%), Pantoea_sp._Cy-640 (2.38%), unclassified_g_Lactococcus (1.75%)*	*Weissella cibaria (88.78%), unclassified_g_Enterobacter (3.25%), unclassified_f_Enterobacteriaceae (2.98%), unclassified_g_Leuconostoc (1.47%)*
	putrescine-pyruvate transaminase	2.6.1.113	*Burkholderia gladioli (100.00%)*	*Burkholderia gladioli (76.58%), unclassified_g_Burkholderia (23.42%)*
	putrescine carbamoyltransferase	2.1.3.6	*Lactococcus lactis (100.00%)*	*Lactococcus lactis (100.00%)*
	spermine oxidase	1.5.3.16	-	*Aspergillus niger (52.16%), Aspergillus welwitschiae (47.84%)*
	aldehyde dehydrogenase (NAD+)	1.2.1.3	*Burkholderia gladioli (69.80%), unclassified_g_Burkholderia (22.66%), Pantoea dispersa (2.65%), Enterobacter asburiae (1.20%), Saccharomyces cerevisiae (1.05%)*	*Saccharomyces cerevisiae (35.33%), Aspergillus niger (31.72%), Aspergillus phoenicis (10.14%), unclassified_g_Burkholderia (5.82%), Burkholderia gladioli (4.47%), Enterobacter asburiae (3.94%), Monascus purpureus (2.20%), Pantoea ananatis (1.18%), unclassified_g_Enterobacter (1.02%), Bacillus amyloliquefaciens (1.01%)*
Amino acid synthesis	histidinol dehydrogenase	1.1.1.23	*Burkholderia gladioli (55.67%), unclassified_g_Pantoea (25.64%), Klebsiella pneumoniae (4.41%), Leuconostoc lactis (3.31%), Kosakonia_sp._CCTCC_M2018092 (2.49%), Enterobacter asburiae (2.19%),*	*Aspergillus niger (41.27%), Saccharomyces cerevisiae (18.72%), Leuconostoc lactis (15.96%), Kosakonia_sp._CCTCC_M2018092 (5.91%), Enterobacter asburiae (4.69%), Burkholderia gladioli (3.47%), Monascus purpureus (1.69%), Enterobacter bugandensis*

			Enterobacter cloacae (1.59%), Lactococcus lactis (1.39%)	*(1.65%), Bacillus siamensis (1.34%), Enterobacter cloacae (1.24%)*
	peptidase A	3.4.13.18	-	-
	histidinol phosphate phosphatase	3.1.3.15	*unclassified_g_Pantoea (42.97%), Leuconostoc lactis (18.64%), unclassified_g_Klebsiella (9.98%), Leuconostoc citreum (9.59%), unclassified_f_Enterobacteriaceae (6.32%), unclassified_g_Enterobacter (3.62%), Lactococcus lactis (2.78%), Saccharomyces cerevisiae (2.04%), unclassified_g_Cronobacter (1.16%)*	*Leuconostoc lactis (43.48%), Saccharomyces cerevisiae (21.90%), Aspergillus niger (14.80%), unclassified_f_Enterobacteriaceae (6.65%), unclassified_g_Enterobacter (4.23%), Leuconostoc citreum (2.59%), unclassified_g_Pantoea (1.29%), Bacillus amyloliquefaciens (1.13%), Lactococcus lactis (1.11%)*
	prephenate dehydrogenase (NADP+)	1.3.1.13	*Saccharomyces cerevisiae (83.33%), Monascus purpureus (16.67%)*	*Aspergillus niger (52.77%), Saccharomyces cerevisiae (45.41%), Monascus purpureus (1.77%)*
	arogenate dehydrogenase	1.3.1.43	*Klebsiella pneumoniae (100%)*	*Klebsiella pneumoniae (100.00%)*
	lysine-2-oxoglutarate reductase	1.5.1.8	-	-
	phenylalaninase	1.14.16.1	*Burkholderia gladioli (100%)*	*Burkholderia gladioli (99.50%)*
	histidinol-phosphate transaminase	2.6.1.9	*Burkholderia gladioli (71.25%), unclassified_g_Pantoea (15.54%), unclassified_f_Enterobacteriaceae (2.76%), Leuconostoc citreum (2.47%), Leuconostoc lactis (2.32%), Escherichia coli (1.47%)*	*Aspergillus phoenicis (22.37%), Leuconostoc lactis (20.80%), Saccharomyces cerevisiae (18.94%), Burkholderia gladioli (10.53%), Escherichia coli (5.23%), unclassified_g_Burkholderia (4.26%), Enterobacter cloacae (2.74%), Leuconostoc*

				citreum (2.45%), Enterobacter bugandensis (2.08%), unclassified_g_Enterobacter (1.68%), unclassified_g_Pantoea (1.47%), Bacillus amyloliquefaciens (1.29%), Enterobacter asburiae (1.19%), Burkholderia_sp._Bp8992 (1.08%), Pantoea deleyi (1.06%), Monascus purpureus (1.00%)
	aromatic-amino-acid transaminase	2.6.1.57	*Burkholderia gladioli (36.12%), unclassified_g_Burkholderia (34.04%), Pantoea_sp._Ap-959 (17.60%), Klebsiella pneumoniae (2.80%), Klebsiella quasipneumoniae (2.36%), unclassified_g_Enterobacter (1.56%)*	*Monascus purpureus (33.55%), Saccharomyces cerevisiae (25.29%), unclassified_g_Burkholderia (12.94%), Kosakonia cowanii (4.98%), unclassified_g_Enterobacter (4.54%), Enterobacteriaceae_bacterium_RIT693 (4.25%), Enterobacter asburiae (3.11%), Pantoea ananatis (2.32%), Enterobacter cloacae (2.13%), Cedecea lapagei (2.01%), Pantoea_sp. (1.66%)*
	tyrosine transaminase	2.6.1.5	*Saccharomyces cerevisiae (82.63%), Monascus purpureus (11.80%), Aspergillus awamori (4.75%)*	*Monascus purpureus (56.04%), Saccharomyces cerevisiae (42.25%), Aspergillus niger (1.09%)*
	phenylalanine(histidine) transaminase	2.6.1.58	*Saccharomyces cerevisiae (98.57%), [Candida] glabrata (1.43%)Saccharomyces cerevisiae (83.33%), Monascus purpureus (16.67%)*	*Saccharomyces cerevisiae (99.92%)*
	L-glutamate gamma-semialdehyde dehydrogenase	1.2.1.88	*Burkholderia gladioli (52.24%), Pantoea dispersa (23.42%), unclassified_g_Enterobacter (7.49%), Klebsiella pneumoniae (4.63%), Kosakonia cowanii (3.94%), Enterobacter kobei (1.92%), Enterobacter_sp._BWH52 (1.62%)*	*Aspergillus niger (31.47%), Aspergillus welwitschiae (23.90%), unclassified_g_Enterobacter (14.61%), Saccharomyces cerevisiae (6.57%), Kosakonia cowanii (6.01%), Enterobacter kobei (3.52%), Burkholderia gladioli (3.37%),*

				Enterobacter_sp._BWH52 (3.00%), Monascus purpureus (1.73%), Bacillus amyloliquefaciens (1.04%)
	glutamate synthase (NADPH)	1.4.1.13	*unclassified_g_Burkholderia (49.75%), Pantoea dispersa (15.36%), Burkholderia pseudomallei (6.90%), Klebsiella pneumoniae (5.12%), Enterobacter sichuanensis (4.29%), unclassified_g_Enterobacter (3.62%), Klebsiella_cf._planticola_B43 (2.99%), Kosakonia cowanii (2.45%), Enterobacter roggenkampii (1.85%), Salmonella enterica (1.62%), Lactococcus lactis (1.33%)*	*unclassified_g_Enterobacter (30.39%), Klebsiella_cf._planticola_B43 (11.88%), Kosakonia cowanii (10.26%), unclassified_g_Burkholderia (7.59%), Enterobacter roggenkampii (6.22%), Klebsiella oxytoca (3.80%), Klebsiella pneumoniae (3.75%), Bacillus amyloliquefaciens (3.70%), Pantoea deleyi (3.08%), Salmonella enterica (2.58%), Enterobacter_sp._MGH_22 (2.54%), Lactococcus lactis (2.01%), unclassified_g_Pantoea (1.82%), Cronobacter dublinensis (1.46%), Pantoea stewartii (1.38%), Burkholderia pseudomallei (1.01%)*
	glutamate synthase (NADH)	1.4.1.14	*Saccharomyces cerevisiae (68.77%), Monascus purpureus (30.79%)*	*Aspergillus nomiae (61.94%), Saccharomyces cerevisiae (34.01%), Monascus purpureus (3.11%)*
	glutamate dehydrogenase	1.4.1.2	*Saccharomyces cerevisiae (62.98%), Monascus purpureus (32.80%), Rhodococcus erythropolis (2.11%), Rhodococcus_sp._ADH (2.11%)*	*Aspergillus eucalypticola (54.98%), Saccharomyces cerevisiae (34.91%), Monascus purpureus (3.15%), Bacillus amyloliquefaciens (2.98%), Burkholderia arboris (2.03%)*
	glutamate dehydrogenase [NAD(P)+]	1.4.1.3	*Burkholderia plantarii (93.25%), Klebsiella aerogenes (5.42%)*	*Burkholderia plantarii (61.19%), unclassified_g_Pantoea (16.13%), Pantoea_sp._ARC607 (8.73%), unclassified_g_Burkholderia (4.18%), Klebsiella aerogenes (2.13%),*

				Chitinophagaceae_bacterium_YG09 (2.06%), Pantoea agglomerans (1.93%), Ferruginibacter_sp. (1.70%), Saccharopolyspora hirsuta (1.68%)
	glutamate dehydrogenase (NADP+)	1.4.1.4	*unclassified_f_Enterobacteriaceae (28.80%), Kosakonia cowanii (20.28%), Saccharomyces cerevisiae (12.09%), Enterobacter roggenkampii (10.87%), Enterobacter asburiae (8.74%), unclassified_g_Enterobacter (6.72%), Franconibacter pulveris (6.08%), Enterobacter cloacae (3.60%), Monascus purpureus (1.95%)*	*Saccharomyces cerevisiae (41.44%), Monascus purpureus (37.45%), Kosakonia cowanii (6.86%), Enterobacter roggenkampii (4.90%), Enterobacter asburiae (4.19%), unclassified_g_Enterobacter (2.66%), Enterobacter cloacae (1.41%)*
	glutaminase	3.5.1.2	*unclassified_g_Burkholderia (54.56%), Pantoea dispersa (22.52%), Weissella cibaria (10.07%), Klebsiella pneumoniae (3.82%), Leuconostoc citreum (2.85%), unclassified_g_Enterobacter (2.18%), Kosakonia_sp._CCTCC_M2018092 (1.72%)*	*Weissella cibaria (51.68%), unclassified_g_Burkholderia (9.77%), Kosakonia_sp._CCTCC_M2018092 (8.99%), Bacillus amyloliquefaciens (7.97%), unclassified_g_Enterobacter (7.78%), Leuconostoc citreum (3.92%), unclassified_g_Pantoea (2.59%), Burkholderia_sp._Bp8992 (1.68%), Leuconostoc holzapfelii (1.42%), Lactococcus lactis (1.25%)*
	glutaminase-asparaginase	3.5.1.38	-	*Burkholderia gladioli (97.81%), Variovorax_sp._SCN_67-85 (2.19%)*
	N-formylglutamate deformylase	3.5.1.68	*Burkholderia gladioli (82.90%), unclassified_g_Pantoea (16.44%)*	*Burkholderia gladioli (70.12%), Pantoea ananatis (12.10%), Pantoea deleyi (11.80%), Burkholderia territorii (5.26%)*
	succinylglutamate desuccinylase	3.5.1.96	*Burkholderia gladioli (55.58%), Pantoea dispersa (27.94%), Klebsiella pneumoniae (5.14%), unclassified_g_Klebsiella (2.39%),*	*Burkholderia gladioli (23.42%), Enterobacter asburiae (14.10%), Kosakonia_sp._CCTCC_M2018092 (12.23%),*

			unclassified_g_Enterobacter (1.39%), Enterobacter asburiae (1.22%)	*Enterobacter kobei (11.74%), Enterobacter bugandensis (9.39%), Enterobacter cloacae (6.78%), Pantoea deleyi (6.55%), unclassified_g_Pantoea (4.42%), Burkholderia_sp._Bp8992 (3.56%), Enterobacter ludwigii (2.24%), Enterobacter hormaechei (2.15%), Pantoea ananatis (1.50%)*
	5-oxoprolinase (ATP-hydrolysing)	3.5.2.9	*Burkholderia gladioli (64.80%), unclassified_g_Burkholderia (12.15%), unclassified_g_Pantoea (5.78%), Pantoea endophytica (4.10%), Enterobacteriaceae bacterium RIT692 (4.06%), Enterobacter cloacae (1.28%), unclassified_g_Klebsiella (1.18%), unclassified_g_Enterobacter (1.16%)*	*Aspergillus niger (31.33%), Monascus purpureus (17.07%), Saccharomyces cerevisiae (9.63%), Aspergillus flavus (9.04%), Aspergillus awamori (7.26%), Burkholderia gladioli (6.89%), unclassified_g_Enterobacter (3.96%), Enterobacter cloacae (3.56%), Kosakonia cowanii (1.59%), unclassified_g_Burkholderia (1.47%), Enterobacter asburiae (1.14%), Pantoea ananatis (1.13%)*
	formimidoylglutamase	3.5.3.8	*Klebsiella pneumoniae (36.79%), Enterobacter asburiae (35.96%), Kosakonia cowanii (12.39%), Enterobacter cloacae (8.20%), Enterobacter_sp._18A13 (2.33%), unclassified_g_Enterobacter (2.06%), Enterobacter kobei (1.03%)*	*unclassified_g_Enterobacter (53.21%), Kosakonia cowanii (20.31%), Enterobacter asburiae (13.70%), Bacillus amyloliquefaciens (2.97%), Enterobacter kobei (2.76%), Enterobacter_sp._18A13 (2.35%), Enterobacter ludwigii (1.77%), Enterobacter_sp._NFIX59 (1.53%)*
	acetylornithine deacetylase	3.5.1.16	*Burkholderia gladioli (40.73%), Pantoea dispersa (19.98%), unclassified_g_Burkholderia (18.75%), unclassified_g_Pantoea (9.43%), Klebsiella pneumoniae (3.22%), unclassified_f_Enterobacteriaceae (1.79%),*	*Aspergillus niger (32.46%), Leclercia adecarboxylata (12.30%), unclassified_f_Enterobacteriaceae (12.08%), Burkholderia gladioli (11.11%), Klebsiella pneumoniae (7.04%), unclassified_g_Burkholderia (6.52%), Pantoea_sp._JKS000250 (3.16%), Pantoea*

			Leclercia adecarboxylata (1.32%), unclassified_g_Enterobacter (1.15%)	*ananatis (2.79%), Enterobacter cloacae (2.73%), Klebsiella oxytoca (2.23%), Pantoea_sp._OXWO6B1 (1.71%), Monascus purpureus (1.70%), Lactococcus lactis (1.57%)*
	glutamate N-acetyltransferase	2.3.1.35	*Weissella cibaria (60.91%), Burkholderia gladioli (32.81%), Leuconostoc citreum (2.53%), Leuconostoc lactis (2.21%)*	*Weissella cibaria (80.56%), Leuconostoc lactis (7.19%), Saccharomyces cerevisiae (5.42%), Aspergillus niger (3.06%), Burkholderia gladioli (1.47%)*
	arginase	3.5.3.1	*Klebsiella pneumoniae (57.01%), Saccharomyces cerevisiae (33.98%), Aspergillus niger (6.33%), Escherichia coli (1.19%)*	*Monascus purpureus (37.35%), Aspergillus niger (30.82%), Saccharomyces cerevisiae (29.06%), Bacillus amyloliquefaciens (2.09%)*
	membrane alanyl aminopeptidase	3.4.11.2	*Weissella cibaria (40.67%), Burkholderia gladioli (17.48%), unclassified_g_Burkholderia (13.56%), Weissella confusa (10.36%), Pantoea dispersa (8.37%), Weissella cibaria (1.70%), unclassified_f_Enterobacteriaceae (1.58%)*	*Weissella cibaria (78.71%), Weissella confusa (6.15%), Leuconostoc lactis (5.14%), Kosakonia cowanii (2.76%), unclassified_g_Enterobacter (1.27%), Burkholderia gladioli (1.14%)*
	aspartate transaminase	2.6.1.1	*Burkholderia gladioli (45.69%), unclassified_g_Pantoea (21.44%), Type-C_symbiont_of_Plautia_stali (16.00%), Pantoea dispersa (5.18%), unclassified_g_Enterobacter (3.55%), unclassified_g_Kosakonia (3.03%), Saccharomyces cerevisiae (1.19%), Enterobacter cloacae (1.05%)*	*Saccharomyces cerevisiae (28.08%), Aspergillus luchuensis (21.82%), Aspergillus neoniger (18.17%), unclassified_g_Enterobacter (7.57%), unclassified_g_Kosakonia (6.43%), Monascus purpureus (4.42%), Burkholderia gladioli (2.92%), Bacillus amyloliquefaciens (2.08%), Aspergillus eucalypticola (2.03%), Shigella sonnei (1.79%), Enterobacter cloacae (1.35%), Enterobacter hormaechei (1.31%)*

	histidinol-phosphate transaminase	2.6.1.9	*Burkholderia gladioli (71.25%), unclassified_g_Pantoea (15.54%), unclassified_f_Enterobacteriaceae (2.76%), Leuconostoc citreum (2.47%), Leuconostoc lactis (2.32%), Escherichia coli (1.47%)*	*Aspergillus phoenicis (22.37%), Leuconostoc lactis (20.80%), Saccharomyces cerevisiae (18.94%), Burkholderia gladioli (10.53%), Escherichia coli (5.23%), unclassified_g_Burkholderia (4.26%), Enterobacter cloacae (2.74%), Leuconostoc citreum (2.45%), Enterobacter bugandensis (2.08%), unclassified_g_Enterobacter (1.68%), unclassified_g_Pantoea (1.47%), Bacillus amyloliquefaciens (1.29%), Enterobacter asburiae (1.19%), Burkholderia_sp._Bp8992 (1.08%), Pantoea deleyi (1.06%), Monascus purpureus (1.00%)*
	tyrosine transaminase	2.6.1.5	*Saccharomyces cerevisiae (82.63%), Monascus purpureus (11.80%), Aspergillus awamori (4.75%)*	*Monascus purpureus (56.04%), Saccharomyces cerevisiae (42.25%), Aspergillus niger (1.09%)*
	tryptophan-phenylpyruvate transaminase	2.6.1.28	*Saccharomyces cerevisiae (98.57%), [Candida] glabrata (1.43%)Burkholderia gladioli (59.83%),*	*Saccharomyces cerevisiae (99.92%)*
	prephenate dehydratase	4.2.1.51	*Burkholderia gladioli (59.83%), Pantoea_sp._GL120224-02 (13.26%), Pantoea dispersa (13.02%), unclassified_f_Enterobacteriaceae (3.10%), Klebsiella pneumoniae (3.07%), Kosakonia cowanii (1.68%), Enterobacter cloacae (1.09%)*	*Saccharomyces cerevisiae (39.04%), Burkholderia gladioli (13.42%), unclassified_f_Enterobacteriaceae (9.99%), Enterobacter cloacae (7.95%), Monascus purpureus (7.54%), unclassified_g_Enterobacter (4.72%), Pantoea ananatis (3.84%), Pantoea deleyi (2.60%), Pantoea agglomerans (2.53%), Enterobacter quasihormaechei (2.07%), Lactococcus lactis (1.68%), unclassified_g_Burkholderia (1.26%), Bacillus amyloliquefaciens (1.17%)*

	chorismate mutase	5.4.99.5	*Burkholderia gladioli (50.24%), unclassified_g_Pantoea (13.65%), Pantoea_sp._GL120224-02 (11.78%), Pantoea dispersa (8.34%), Kosakonia cowanii (2.86%), unclassified_f_Enterobacteriaceae (2.76%), Klebsiella pneumoniae (1.75%), Enterobacter cloacae (1.63%), Enterobacter kobei (1.16%), Klebsiella variicola (1.01%)*	*Saccharomyces cerevisiae (18.24%), Kosakonia cowanii (14%), Aspergillus niger (13.34%), Burkholderia gladioli (11.06%), Enterobacter cloacae (9.63%), Enterobacter kobei (7.73%), unclassified_g_Enterobacter (3.67%), Pantoea ananatis (3.05%),Pantoea deleyi (2.95%), Bacillus amyloliquefaciens (2.76%), unclassified_g_Pantoea (2.22%), Enterobacter asburiae (2.07%), Enterobacter quasihormaechei (1.61%), Lactococcus lactis (1.26%), Enterobacter oligotrophica (1.03%)*
	arogenate dehydratase	4.2.1.91	*Burkholderia gladioli (60.93%), Pantoea dispersa (30.17%), Klebsiella pneumoniae (7.12%), Pantoea ananatis (1.07%)*	*Burkholderia gladioli (52.11%), Pantoea agglomerans (24.16%), Pantoea ananatis (17.05%), Burkholderia seminalis (4.16%), Klebsiella pneumoniae (1.96%)*
	phenylalanine ammonia-lyase	4.3.1.24	-	*Aspergillus niger (71.89%), Aspergillus phoenicis (27.98%)*
	saccharopine dehydrogenase (NAD+, L-lysine-forming)	1.5.1.7	*Saccharomyces cerevisiae (97.39%), Monascus purpureus (2.61%)*	*Aspergillus phoenicis (52.31%), Saccharomyces cerevisiae (47.47%)*
	diaminopimelate decarboxylase	4.1.1.20	*Weissella cibaria (41.74%), Burkholderia gladioli (30.39%), unclassified_g_Pantoea (14.32%), unclassified_f_Enterobacteriaceae (3.25%), Enterobacter cloacae (2.65%), Leuconostoc citreum (1.88%), Leuconostoc lactis (1.59%)*	*Weissella cibaria (78.65%), Leuconostoc lactis (8.61%), Enterobacter cloacae (3.56%), Enterobacter asburiae (2.05%), unclassified_g_Enterobacter (2.00%), Burkholderia gladioli (1.64%)*
	aromatic-amino-acid transaminase	2.6.1.57	*Burkholderia gladioli (36.12%), unclassified_g_Burkholderia (34.04%), Pantoea_sp._Ap-959 (17.60%), Klebsiella pneumoniae (2.80%), Klebsiella*	*Monascus purpureus (33.55%), Saccharomyces cerevisiae (25.29%), unclassified_g_Burkholderia (12.94%), Kosakonia cowanii (4.98%),*

			quasipneumoniae (2.36%), unclassified_g_Enterobacter (1.56%)	*unclassified_g_Enterobacter (4.54%), Enterobacteriaceae_bacterium_RIT693 (4.25%), Enterobacter asburiae (3.11%), Pantoea ananatis (2.32%), Enterobacter cloacae (2.13%), Cedecea lapagei (2.01%), Pantoea_sp. (1.66%)*
	phenylalanine(histidine) transaminase	2.6.1.58	*Saccharomyces cerevisiae (98.57%), [Candida] glabrata (1.43%)Saccharomyces cerevisiae (83.33%), Monascus purpureus (16.67%)*	*Saccharomyces cerevisiae (99.92%)*
	saccharopine dehydrogenase (NAD+, L-lysine-forming)	1.5.1.7	*Saccharomyces cerevisiae (97.39%), Monascus purpureus (2.61%)*	*Aspergillus phoenicis (52.31%), Saccharomyces cerevisiae (47.47%)*
	diaminopimelate decarboxylase	4.1.1.20	*Weissella cibaria (41.74%), Burkholderia gladioli (30.39%), unclassified_g_Pantoea (14.32%), unclassified_f_Enterobacteriaceae (3.25%), Enterobacter cloacae (2.65%), Leuconostoc citreum (1.88%), Leuconostoc lactis (1.59%)*	*Weissella cibaria (78.65%), Leuconostoc lactis (8.61%), Enterobacter cloacae (3.56%), Enterobacter asburiae (2.05%), unclassified_g_Enterobacter (2.00%), Burkholderia gladioli (1.64%)*
	tryptophan synthase	4.2.1.20	*unclassified_g_Burkholderia (37.15%), Burkholderia gladioli (31.11%), Pantoea dispersa (12.55%), unclassified_g_Pantoea (5.86%), Enterobacter ludwigii (2.92%), Klebsiella pneumoniae (2.75%), Leuconostoc lactis (2.02%), Kosakonia cowanii (1.51%), unclassified_g_Enterobacter (1.07%), unclassified_f_Enterobacteriaceae (1.04%)*	*Aspergillus welwitschiae (27.51%), Aspergillus niger (26.57%), Saccharomyces cerevisiae (15.57%), Enterobacter ludwigii (5.01%), Kosakonia cowanii (4.11%), unclassified_g_Burkholderia (3.90%), unclassified_g_Enterobacter (3.61%), Burkholderia gladioli (2.94%), unclassified_f_Enterobacteriaceae (2.55%), Bacillus amyloliquefaciens (1.78%), Monascus purpureus (1.70%), Leuconostoc lactis (1.52%), Enterobacter roggenkampii (1.01%)*

	alanine dehydrogenase	1.4.1.1	*Lactococcus garvieae (59.60%), Pantoea ananatis (31.46%), Burkholderia ubonensis (8.94%)*	*Lactococcus garvieae (34.83%), Pantoea allii (27.27%), Bacillus amyloliquefaciens (14.57%), unclassified_g_Bacillus_f_Bacillaceae (12.10%), Bacillus ginsengihumi (8.90%), Saccharopolyspora dendranthemae (1.69%)*
Amino acid degradation	histidine ammonia-lyase	4.3.1.3	*Pantoea dispersa (30.85%), unclassified_g_Burkholderia (30.64%), Burkholderia gladioli (27.26%), unclassified_g_Pantoea (4.64%), Klebsiella pneumoniae (3.17%), Klebsiella_cf._planticola_B43 (2.36%)*	*unclassified_g_Enterobacter (30.70%), Klebsiella_cf._planticola_B43 (22.38%), unclassified_g_Burkholderia (13.60%), Burkholderia gladioli (11.43%), unclassified_g_Pantoea (7.25%), Bacillus amyloliquefaciens (5.34%), Pantoea_sp._AG702 (2.04%), Pantoea_sp._ARC607 (1.82%), Lelliottia_sp._WB101 (1.13%), Burkholderia_sp._Bp8992 (1.08%)*
	L-histidine Nalpha-methyltransferase	2.1.1.44	*Monascus purpureus (100%)*	*Monascus purpureus (99.86%)*
	tyrosinase	1.14.18.1	*Aspergillus niger (69.89%), Monascus purpureus (30.11%)Saccharomyces cerevisiae (82.51%),*	*Aspergillus niger (57.66%), Monascus purpureus (27.18%), Aspergillus awamori (14.82%)*
	amino-acid N-acetyltransferase	2.3.1.1	*Burkholderia gladioli (44.58%), Weissella cibaria (43.98%), unclassified_g_Pantoea (2.81%), Leuconostoc citreum (1.82%), Leuconostoc lactis (1.60%), Cronobacter malonaticus (1.09%), unclassified_g_Enterobacter (1.08%)*	*Weissella cibaria (67.22%), Saccharomyces cerevisiae (11.49%), Leuconostoc lactis (6.00%), Aspergillus phoenicis (5.51%), Aspergillus niger (2.55%), Burkholderia gladioli (2.28%), unclassified_g_Enterobacter (1.17%)*
	carbamyl phosphate synthetase (glutamine)	6.3.5.5	*Weissella cibaria (64.37%), unclassified_g_Weissella (11.74%), unclassified_g_Burkholderia (10.46%), unclassified_g_Pantoea (2.64%), Leuconostoc*	*Weissella cibaria (71.44%), unclassified_g_Weissella (12.02%), Aspergillus niger (5.38%), Saccharomyces cerevisiae*

			citreum (1.83%), Leuconostoc lactis (1.47%), Kosakonia cowanii (1.31%), Klebsiella pneumoniae (1.27%)	*(3.45%), Leuconostoc lactis (3.41%), Kosakonia cowanii (1.03%)*
	glutamate 5-kinase	2.7.2.11	*unclassified_g_Burkholderia (47.92%), unclassified_g_Pantoea (25.61%), unclassified_f_Enterobacteriaceae (4.37%), Klebsiella quasipneumoniae (4.11%), Leuconostoc citreum (3.93%), Leuconostoc lactis (3.83%), Enterobacter roggenkampii (3.50%), Saccharomyces cerevisiae (1.65%), Lactococcus lactis (1.65%), Cronobacter sakazakii (1.09%)*	*Saccharomyces cerevisiae (35.19%), Leuconostoc lactis (20.90%), Aspergillus niger (17.81%), unclassified_f_Enterobacteriaceae (7.80%), Enterobacter roggenkampii (6.74%), unclassified_g_Burkholderia (3.37%), Leuconostoc citreum (1.73%), Bacillus amyloliquefaciens (1.37%), Pantoea deleyi (1.06%)*
	D-amino-acid oxidase	1.4.3.3	*Monascus purpureus (79.56%), Aspergillus tanneri (18.67%), Saccharopolyspora spinosa (1.78%)*	*Aspergillus niger (84.75%), Monascus purpureus (9.25%), Aspergillus tanneri (4.31%)*
	ornithine aminotransferase	2.6.1.13	*Saccharomyces cerevisiae (82.51%), Monascus purpureus (16.67%)*	*Aspergillus niger (35.94%), Aspergillus welwitschiae (34.17%), Saccharomyces cerevisiae (26.89%), Monascus purpureus (1.34%), Bacillus amyloliquefaciens (1.24%)*
	D-amino-acid oxidase	1.4.3.3	*Monascus purpureus (79.56%), Aspergillus tanneri (18.67%), Saccharopolyspora spinosa (1.78%)*	*Aspergillus niger (84.75%), Monascus purpureus (9.25%), Aspergillus tanneri (4.31%)*
	glycine oxidase	1.4.3.19	*Burkholderia gladioli (69.86%), unclassified_g_Pantoea (29.45%)*	*Burkholderia gladioli (46.07%), Pantoea_sp._Sc1 (17.22%), Bacillus amyloliquefaciens (15.68%), unclassified_g_Pantoea (15.01%), Bacillus ginsengihumi (2.46%), Burkholderia seminalis (2.06%)*

	glycine dehydrogenase (aminomethyl-transferring)	1.4.4.2	*Burkholderia gladioli (46.20%), Pantoea dispersa (30.84%), Klebsiella pneumoniae (4.40%), Kosakonia_sp._CCTCC_M2018092 (3.13%), Enterobacter cloacae (2.92%), Enterobacter hormaechei (2.90%), Kosakonia cowanii (2.16%), unclassified_g_Enterobacter (1.70%), Cronobacter malonaticus (1.14%)*	*Aspergillus niger (29.31%), Saccharomyces cerevisiae (22.61%), Kosakonia_sp._CCTCC_M2018092 (8.04%), unclassified_g_Enterobacter (7.29%), Lupinus albus (6.92%), Kosakonia cowanii (5.89%), Burkholderia gladioli (4.78%), Pantoea deleyi (2.96%), Enterobacter cloacae (2.29%), Bacillus amyloliquefaciens (2.05%), unclassified_f_Enterobacteriaceae (1.60%), Monascus purpureus (1.40%)*
	glycine C-acetyltransferase	2.3.1.29	*Burkholderia gladioli (59.74%), unclassified_g_Pantoea (28.37%), Kosakonia pseudosacchari (3.16%), unclassified_g_Enterobacter (2.80%), Klebsiella pneumoniae (2.00%), Enterobacter bugandensis (1.85%), Enterobacter roggenkampii (1.33%)*	*Kosakonia cowanii (23.59%), unclassified_g_Enterobacter (21.96%), Burkholderia gladioli (14.96%), Enterobacter bugandensis (11.21%), Enterobacter roggenkampii (9.37%), unclassified_g_Pantoea (4.64%), unclassified_g_Burkholderia (3.55%), Lactococcus garvieae (3.10%), Bacillus amyloliquefaciens (2.79%), Pantoea agglomerans (2.05%)*
	5-aminolevulinate synthase	2.3.1.37	*Monascus purpureus (54.33%), Saccharomyces cerevisiae (43.73%), Pantoea ananatis (1.94%)*	*Aspergillus niger (57.09%), Saccharomyces cerevisiae (31.17%), Monascus purpureus (10.93%)*
	D-amino-acid N-acetyltransferase	2.3.1.36	*Saccharomyces cerevisiae (100%)*	*Saccharomyces cerevisiae (100.00%)*
	saccharopine dehydrogenase (NAD+, L-lysine-forming)	1.5.1.7	*Saccharomyces cerevisiae (97.39%), Monascus purpureus (2.61%)*	*Aspergillus phoenicis (52.31%), Saccharomyces cerevisiae (47.47%)*
	lysine 2-monooxygenase	1.13.12.2	-	-

	tryptophanase	1.13.11.11	*Burkholderia gladioli (100%)*	*Burkholderia gladioli (81.76%), Burkholderia_sp._Bp8992 (18.24%)*
	tryptophan transaminase	2.6.1.27	*Saccharomyces cerevisiae (82.63%), Monascus purpureus (11.80%), Aspergillus niger (4.75%)*	*Monascus purpureus (56.04%), Saccharomyces cerevisiae (42.25%), Aspergillus niger (1.09%)*
	tryptophanase	4.1.99.1	*Pantoea ananatis (94.30%), Cronobacter dublinensis (4.56%), Klebsiella quasipneumoniae (1.14%)Weissella cibaria (40.67%), Burkholderia gladioli (17.48%),*	*Pantoea ananatis (94.52%), Klebsiella quasipneumoniae (5.48%)*
	D-amino-acid oxidase	1.4.3.3	*Monascus purpureus (79.56%), Aspergillus tanneri (18.67%), Saccharopolyspora spinosa (1.78%)*	*Aspergillus niger (84.75%), Monascus purpureus (9.25%), Aspergillus tanneri (4.31%)*
	glycine amidinotransferase	2.1.4.1	-	-
	arginine N-succinyltransferase	2.3.1.109	*Burkholderia gladioli (37.70%), unclassified_g_Burkholderia (35.81%), Pantoea dispersa (15.21%), unclassified_g_Klebsiella (3.23%), Klebsiella pneumoniae (2.80%), unclassified_f_Enterobacteriaceae (2.36%)*	*unclassified_f_Enterobacteriaceae (30.52%), unclassified_g_Burkholderia (22.36%), Burkholderia gladioli (19.23%), Enterobacter huaxiensis (9.32%), unclassified_g_Enterobacter (6.31%), Pantoea deleyi (3.94%), unclassified_g_Pantoea (3.81%), Burkholderia cepacia (2.22%)*
	arginine deiminase	3.5.3.6	*Weissella cibaria (92.31%), Enterobacter_sp._SES19 (4.59%), unclassified_g_Lactococcus (1.64%)*	*Weissella cibaria (94.98%), Escherichia coli (3.84%)*
	nitric-oxide synthase (flavodoxin)	1.14.14.47	*Bacillus ginsengihumi (100.00%)*	*Bacillus amyloliquefaciens (87.44%), Bacillus ginsengihumi (9.06%), Saccharopolyspora shandongensis (2.44%), unclassified_g_Staphylococcus (1.07%)*
	D-arginine dehydrogenase	1.4.99.6	-	*Burkholderia gladioli (86.67%), Burkholderia cepacia (13.33%)*

Table S3. Microbial contributions to flavor metabolizing enzymes in GTW and WYW brewing.

Enzyme function	Enzyme name	Enzyme number	GTW	WYW
			Distribution of microbes (>1%)	Distribution of microbes (>1%)
Glycolysis	glycogen phosphorylase	2.4.1.1	*Weissella cibaria (57.67%), Pantoea dispersa (13.96%), Pantoea_sp._AG1095 (9.04%), unclassified_o_Enterobacterales (4.37%), unclassified_f_Enterobacteriaceae (2.61%), Kosakonia cowanii (2.60%), Klebsiella pneumoniae (2.16%)*	*Weissella cibaria (76.82%), unclassified_o_Enterobacterales (5.73%), Saccharomyces cerevisiae (3.94%), Aspergillus niger (3.32%), Kosakonia cowanii (2.55%), Enterobacter cloacae (1.08%)*
	glucan 1,4-alpha-glucosidase	3.2.1.3	*Saccharomyces cerevisiae (47.18%), Monascus purpureus (38.03%), Pantoea ananatis (35.21%), Byssochlamys spectabilis (9.15%), Rhizopus oryzae (9.15%), Pantoea_sp._OXWO6B1 (8.45%)*	*Aspergillus niger (58.56%), Saccharomyces cerevisiae (30.21%), Monascus purpureus (3.86%), Pantoea ananatis (2.01%), Caballeronia megalochromosomata (1.54%), Trinickia soli (1.39%), Byssochlamys spectabilis (1.08%)*
	amylo-alpha-1,6-glucosidase	3.2.1.33	*Saccharomyces cerevisiae (84.96%), Monascus purpureus (15.04%),*	*Monascus purpureus (55.31%), Saccharomyces cerevisiae (44.35%)*
	glucose-1-phosphatase	3.1.3.10	*unclassified_g_Pantoea (66.36%)， Klebsiella pneumoniae (8.14%)， Enterobacter cloacae (4.36%)， Klebsiella_cf._planticola_B43 (3.67%)， Kosakonia cowanii (3.40%)，unclassified_f_Enterobacteriaceae (2.72%)*	*Enterobacter cloacae (20.86%)，Klebsiella_cf._planticola_B43 (15.28%)，Kosakonia cowanii (14.42%)， Enterobacter kobei (10.86%)， Enterobacter roggenkampii (6.26%)， Lactococcus lactis (5.09%)，Klebsiella pneumoniae (4.91%)， Type-F_symbiont_of_Plautia_stali (4.42%)，Enterobacter bugandensis (4.36%)， Pantoea ananatis (3.93%)， Pantoea_sp._AMG_501 (3.50%)*

	cyclomaltodextrinase	3.2.1.54	*Leuconostoc lactis (88.21%), Lactococcus garvieae (9.63%)*	*Leuconostoc lactis (96.82%), Lactococcus garvieae (2.49%)*
	isoamylase	3.2.1.68	*Burkholderia gladioli (51.20%)，unclassified_g_Pantoea (33.00%)，Enterobacter_sp._N18-03635 (8.22%)，Kosakonia cowanii (3.28%)*	*Enterobacter_sp._N18-03635 (51.92%)，Kosakonia cowanii (17.89%)， Burkholderia gladioli (11.76%)， Enterobacter kobei (6.37%)， unclassified_g_Pantoea (3.99%)，Enterobacter roggenkampii (3.79%)，unclassified_g_Enterobacter (1.22%)*
	alpha-amylase	3.2.1.1	*Burkholderia gladioli (54.91%), Enterobacter asburiae (7.36%), Klebsiella pneumoniae (6.14%), unclassified_f_Enterobacteriaceae (6.02%), unclassified_g_Enterobacter (4.58%), Kosakonia cowanii (4.53%), Enterobacter bugandensis (4.21%), Lactococcus lactis (2.26%)*	*Aspergillus niger (34.3%), Enterobacter asburiae (16.63%), unclassified_g_Enterobacter (9.06%), Enterobacter bugandensis (8.96%), Kosakonia cowanii (8.04%), Enterobacter_sp._RIT_418 (3.06%), Enterobacter roggenkampii (2.54%), Lactococcus lactis (2.08%)*
	glucan 1,4-alpha-maltohydrolase	3.2.1.133	*Leuconostoc lactis (88.21%), Lactococcus garvieae (9.63%)*	*Leuconostoc lactis (96.82%), Lactococcus garvieae (2.49%)*
	alpha-glucosidase	3.2.1.20	*Weissella cibaria (64.93%)，Pantoea_sp._Ap-870 (11.26%)， Enterobacter asburiae (5.93%)，unclassified_f_Enterobacteriaceae (3.53%)，unclassified_g_Klebsiella (3.24%)，Kosakonia_sp._CCTCC_M2018092 (2.26%)*	*Weissella cibaria (69.45%)， Aspergillus niger (12.94%)， Enterobacter asburiae (6.75%)，Leuconostoc lactis (3.85%)，Kosakonia_sp._CCTCC_M2018092 (2.05%)，Kosakonia cowanii (1.37%)*
	4-alpha-glucanotransferase	2.4.1.25	*Pantoea dispersa (33.99%), Pantoea_sp._BK028 (23.42%), unclassified_f_Enterobacteriaceae (10.01%), Kosakonia cowanii (4.55%), Saccharomyces cerevisiae (4.49%), Enterobacter hormaechei (4.38%), Enterobacter_sp._J49 (2.50%),*	*Monascus purpureus (33.99%), Saccharomyces cerevisiae (27.25%), Enterobacter hormaechei (6.99%), Kosakonia cowanii (5.75%), Enterobacter_sp._J49 (4.41%), Enterobacter cloacae (3.88%), Pantoea ananatis (3.88%), Enterobacter wuhouensis (3.32%), Enterobacter*

			Pantoea ananatis (2.26%), Lactococcus lactis (2.13%)	*asburiae (2.62%), Pantoea deleyi (2.46%), Lactococcus lactis (1.41%)*
	maltose phosphorylase	2.4.1.8	*Weissella cibaria (88.30%), Leuconostoc mesenteroides (6.49%), Leuconostoc lactis (4.61%)*	*Weissella cibaria (91.67%), Leuconostoc lactis (6.11%), Leuconostoc mesenteroides (1.80%)*
	oligo-1,6-glucosidase	3.2.1.10	*Leuconostoc citreum (44.94%), Saccharomyces cerevisiae (28.18%), Leuconostoc lactis (13.97%), Pediococcus damnosus (3.56%), Monascus purpureus (2.70%), Franconibacter pulveris (2.55%), unclassified_g_Cronobacter (1.97%)*	*Saccharomyces cerevisiae (85.75%), Leuconostoc lactis (8.67%), Leuconostoc citreum (2.54%), Monascus purpureus (1.19%)*
	phosphoglucomutase (alpha-D-glucose-1,6-bisphosphate-dependent)	5.4.2.2	*unclassified_g_Weissella (49.85%), unclassified_g_Burkholderia (25.62%), unclassified_g_Pantoea (12.59%), Lelliottia nimipressuralis (3.25%)*	*unclassified_g_Weissella (66.94%), Saccharomyces cerevisiae (11.78%), Aspergillus niger (8.31%), Leuconostoc lactis (4.89%),*
	hexokinase	2.7.1.1	*Saccharomyces cerevisiae (79.56%), Monascus purpureus (20.24%)*	*Saccharomyces cerevisiae (54.9%), Aspergillus niger (26.02%), Aspergillus welwitschiae (9.82%), Uncinocarpus reesii (6.86%), Monascus purpureus (2.15%)*
	glucokinase	2.7.1.2	*unclassified_g_Weissella (41.82%), unclassified_g_Burkholderia (25.92%), Burkholderia gladioli (16.40%), unclassified_g_Pantoea (10.39%), unclassified_f_Enterobacteriaceae (2.79%)*	*unclassified_g_Weissella (91.56%), unclassified_g_Leuconostoc (2.27%), Enterobacter asburiae (1.27%), unclassified_f_Enterobacteriaceae (1.12%)*
	polyphosphate---glucose phosphotransferase	2.7.1.63	*Burkholderia gladioli (98.96%), Pantoea ananatis (1.04%)*	*Burkholderia gladioli (20.14%), Pantoea ananatis (9.20%)*
	glucose-6-phosphate isomerase	5.3.1.9	*Weissella cibaria (51.22%), Burkholderia gladioli (24.13%), unclassified_g_Pantoea (11.06%), Klebsiella pneumoniae (4.13%),*	*Weissella cibaria (73.67%), Leuconostoc lactis (6.13%), Saccharomyces cerevisiae (5.27%), Aspergillus welwitschiae (4.85%), Enterobacter*

			Enterobacter hormaechei (1.57%), Leuconostoc citreum (1.43%), Leuconostoc lactis (1.43%), Klebsiella oxytoca (1.17%)	*hormaechei (2.03%), Klebsiella oxytoca (1.65%), Klebsiella pneumoniae (1.42%)*
	fructose-6-phosphate kinase	2.7.1.11	*Klebsiella pneumoniae (40.25%), unclassified_f_Enterobacteriaceae (22.94%), Saccharomyces cerevisiae (7.93%), unclassified_g_Enterobacter (7.41%), Enterobacter_sp._UCD-UG_FMILLET (5.78%), unclassified_g_Pantoea (2.52%), Enterobacter cloacae (2.22%), Pantoea agglomerans (2.08%), Lactococcus lactis (2.00%)*	*Saccharomyces cerevisiae (43.82%), Aspergillus niger (32.5%), unclassified_f_Enterobacteriaceae (7.77%), unclassified_g_Enterobacter (4.53%), Enterobacter_sp._UCD-UG_FMILLET (4.46%)*
	diphosphate---fructose-6-phosphate 1-phosphotransferase	2.7.1.90	*Rhodococcus erythropolis (50.00%), unclassified_g_Variovorax (50.00%)*	*Rhodococcus erythropolis (75.00%), unclassified_g_Variovorax (25.00%)*
	fructose-bisphosphate aldolase	4.1.2.13	*unclassified_g_Pantoea (50.82%), unclassified_g_Burkholderia (40.77%), Pantoea dispersa (18.17%), unclassified_o_Enterobacterales (12.18%), unclassified_f_Enterobacteriaceae (5.94%), Klebsiella pneumoniae (5.31%), unclassified_g_Enterobacter (4.22%), Leuconostoc lactis (2.14%), Salmonella enterica (2.06%)*	*unclassified_o_Enterobacterales (23.31%), unclassified_f_Enterobacteriaceae (15.81%), Leuconostoc lactis (15.53%), Saccharomyces cerevisiae (13.59%), unclassified_g_Enterobacter (11.85%), Aspergillus welwitschiae (9.39%), Aspergillus niger (8.83%), Salmonella enterica (5.22%), Aspergillus sclerotioniger (2.56%), unclassified_g_Pantoea (2.43%)Klebsiella oxytoca (2.32%)*
	triose-phosphate isomerase	5.3.1.1	*Weissella cibaria (69.56%), Burkholderia gladioli (19.76%), unclassified_f_Enterobacteriaceae (3.30%), Klebsiella pneumoniae (1.50%), Leuconostoc*	*Weissella cibaria (89.52%), Monascus purpureus (2.63%), Saccharomyces cerevisiae (2.58%), Aspergillus neoniger (1.40%)*

			citreum (1.48%), unclassified_g_Pantoea (1.25%)	
	glyceraldehyde-3-phosphate dehydrogenase (phosphorylating)	1.2.1.12	*Weissella cibaria (49.84%), Burkholderia gladioli (27.46%), Pantoea_sp._BK028 (5.01%), Leuconostoc citreum (2.54%), Enterobacter cloacae (1.71%), unclassified_o_Enterobacterales (1.44%), unclassified_f_Enterobacteriaceae (1.39%), Pantoea agglomerans (1.33%), unclassified_g_Enterobacter (1.27%), Enterobacter bugandensis (1.24%), unclassified_g_Lactococcus (1.10%)*	*Weissella cibaria (69.92%), Saccharomyces cerevisiae (10.15%), Aspergillus phoenicis (2.40%), Aspergillus niger (2.13%), Leuconostoc citreum (2.04%), unclassified_o_Enterobacterales (2.03%), Enterobacter cloacae (1.54%), unclassified_g_Enterobacter (1.53%), Enterobacter bugandensis (1.38%)*
	phosphoglycerate kinase	2.7.2.3	*Weissella cibaria (52.15%), unclassified_g_Burkholderia (33.97%), unclassified_g_Pantoea (4.35%), Leuconostoc lactis (2.84%), unclassified_g_Cronobacter (1.76%), unclassified_g_Lactococcus (1.42%), Leuconostoc citreum (1.37%)*	*Weissella cibaria (77.95%), Leuconostoc lactis (8.03%), Saccharomyces cerevisiae (4.72%), Monascus purpureus (3.36%), Enterobacter ludwigii (1.30%), unclassified_g_Cronobacter (1.10%)*
	phosphoglycerate mutase (2,3-diphosphoglycerate-independent)	5.4.2.12	*Kosakonia cowanii (30.70%), Klebsiella pneumoniae (29.20%), Kosakonia oryziphila (6.23%), Cronobacter malonaticus (6.16%), Enterobacter ludwigii (6.04%), unclassified_g_Enterobacter (5.04%), Franconibacter pulveris (4.61%), Enterobacter roggenkampii (4.48%), unclassified_g_Cronobacter (2.99%), Lelliottia nimipressuralis (2.37%)*	*Aspergillus niger (50.29%), Kosakonia cowanii (24.48%), Kosakonia oryziphila (5.97%), Enterobacter ludwigii (4.15%), Enterobacter roggenkampii (2.94%), unclassified_g_Bacillus_f_Bacillaceae (2.67%), Klebsiella pneumoniae (2.10%),*

	phosphoglycerate mutase (2,3-diphosphoglycerate-dependent)	5.4.2.11	*Burkholderia gladioli (34.33%), unclassified_g_Weissella (34.04%), unclassified_g_Pantoea (7.95%), Pantoea_sp._R102 (7.74%), unclassified_f_Enterobacteriaceae (5.98%), Leuconostoc citreum (2.72%)*	*unclassified_g_Weissella (65.21%), Saccharomyces cerevisiae (12.84%), unclassified_g_Leuconostoc (5.14%), unclassified_f_Enterobacteriaceae (4.93%), Leuconostoc lactis (3.23%)*
	phosphopyruvate hydratase	4.2.1.11	*Weissella cibaria (50.33%), unclassified_g_Burkholderia (29.82%), unclassified_f_Enterobacteriaceae (11.64%), unclassified_g_Leuconostoc (2.94%), Lactococcus lactis (1.44%), Saccharomyces cerevisiae (1.16%)*	*Weissella cibaria (69.14%), Saccharomyces cerevisiae (13.16%), unclassified_g_Leuconostoc (7.90%), unclassified_f_Enterobacteriaceae (5.15%)*
	pyruvate kinase	2.7.1.40	*Burkholderia gladioli (40.97%), Weissella cibaria (38.51%), unclassified_g_Pantoea (7.62%), Klebsiella quasipneumoniae (1.66%), Enterobacter_sp._Crenshaw (1.43%), Leuconostoc citreum (1.29%), Pantoea wallisii (1.03%)*	*Weissella cibaria (72.06%), Saccharomyces cerevisiae (8.51%), unclassified_g_Leuconostoc (4.48%), Lichtheimia ramosa (4.42%), Enterobacter_sp._Crenshaw (2.66%), Burkholderia gladioli (1.37%)*
Fatty acids biosynthesis	acetyl-CoA carboxylase	6.4.1.2	*Weissella cibaria (36.01%), Burkholderia gladioli (14.90%), unclassified_g_Burkholderia (13.34%), Weissella_sp._DD23 (12.30%), unclassified_g_Weissella (10.79%), unclassified_g_Pantoea (3.99%), Leuconostoc citreum (1.40%)*	*Weissella cibaria (54.43%), Weissella_sp._DD23 (16.53%), unclassified_g_Weissella (14.79%), Saccharomyces cerevisiae (4.23%), Leuconostoc lactis (2.18%), Lichtheimia ramosa (1.61%), unclassified_g_Leuconostoc (1.43%)*
	Transferring groups other than aminoacyl groups	2.3.1.-	*Burkholderia gladioli (50.21%), unclassified_g_Burkholderia (11.28%), unclassified_g_Pantoea (8.44%), Pantoea dispersa (6.24%), unclassified_f_Enterobacteriaceae (4.76%), unclassified_g_Enterobacter (3.36%),*	*Saccharomyces cerevisiae (35.67%), Aspergillus niger (15.54%), Monascus purpureus (8.27%), unclassified_g_Enterobacter (7.97%), Kosakonia cowanii (7.32%), Aspergillus phoenicis (3.85%), Monascus ruber (3.18%),*

			Pantoea_sp._BK028 (3.19%), Kosakonia cowanii (3.02%), Klebsiella pneumoniae (2.01%), Saccharomyces cerevisiae (1.09%)	*Burkholderia gladioli (2.40%), Enterobacter_sp._KINAN-G (2.28%), unclassified_f_Enterobacteriaceae (1.90%)*
	fatty-acyl-CoA synthase system	2.3.1.86	*Monascus purpureus (36.75%), Lichtheimia ramosa (19.45%), Saccharomyces cerevisiae (19.30%), Monascus pilosus (9.04%), Monascus ruber (9.04%), Aspergillus oryzae (3.06%), Aspergillus niger (1.99%), Rhizopus delemar (1.38%)*	*Aspergillus niger (66.20%), Lichtheimia ramosa (8.81%), Saccharomyces cerevisiae (8.65%), Aspergillus welwitschiae (7.13%), Rhizopus delemar (6.07%), Monascus purpureus (2.11%)*
	[acyl-carrier-protein] S-malonyltransferase	2.3.1.39	*Burkholderia gladioli (43.54%), unclassified_g_Burkholderia (27.80%), Salmonella enterica (12.20%), unclassified_f_Enterobacteriaceae (2.87%), Enterobacter cloacae (2.78%), Leuconostoc lactis (2.15%), Leuconostoc citreum (2.09%), Weissella confusa (1.37%), Klebsiella pneumoniae (1.07%)*	*Leuconostoc lactis (27.31%), Saccharomyces cerevisiae (15.17%), Enterobacter cloacae (8.47%), Aspergillus awamori (7.27%), Weissella confusa (6.70%), Kosakonia cowanii (4.70%), Burkholderia gladioli (4.34%), Salmonella enterica (3.69%), unclassified_g_Enterobacter (3.65%), Pantoea deleyi (3.30%), unclassified_g_Burkholderia (2.91%), unclassified_f_Enterobacteriaceae (2.78%), Leuconostoc citreum (2.50%)*
	beta-ketoacyl-[acyl-carrier-protein] synthase III	2.3.1.180	*Weissella cibaria (49.34%), unclassified_g_Burkholderia (28.02%), Burkholderia gladioli (8.97%), Pantoea dispersa (5.82%), Leuconostoc citreum (2.00%), unclassified_g_Enterobacter (1.31%), Klebsiella pneumoniae (1.07%)*	*Weissella cibaria (86.86%), Leuconostoc lactis (4.40%), unclassified_g_Enterobacter (1.97%), unclassified_g_Leuconostoc (1.86%), Enterobacter_sp._UCD-UG_FMILLET (1.29%)*

	beta-ketoacyl-[acyl-carrier-protein] synthase I	2.3.1.41	*unclassified_g_Pantoea (36.91%), Pantoea dispersa (35.21%), unclassified_f_Enterobacteriaceae (8.75%), unclassified_g_Enterobacter (8.22%), Kosakonia cowanii (3.93%), Enterobacter cloacae (1.91%), Enterobacter roggenkampii (1.46%)*	*unclassified_g_Enterobacter (44.48%), Kosakonia cowanii (16.86%), unclassified_f_Enterobacteriaceae (9.26%), Enterobacter roggenkampii (6.95%), Enterobacter asburiae (6.00%), Pantoea deleyi (4.81%), Pantoea ananatis (3.86%),*
	beta-ketoacyl-[acyl-carrier-protein] synthase II	2.3.1.179	*Weissella cibaria (42.24%), unclassified_g_Burkholderia (25.91%), Type-C_symbiont_of_Plautia_stali (9.07%), unclassified_g_Pantoea (6.99%), unclassified_g_Enterobacter (3.85%), Kosakonia cowanii (3.32%), unclassified_f_Enterobacteriaceae (2.22%), Enterobacter cloacae (1.84%), Leuconostoc citreum (1.43%)*	*Weissella cibaria (71.92%), Saccharomyces cerevisiae (5.01%), Leuconostoc lactis (4.96%), Kosakonia cowanii (4.77%), unclassified_g_Enterobacter (4.77%)*
	3-oxoacyl-[acyl-carrier-protein] reductase	1.1.1.100	*Burkholderia gladioli (36.08%), unclassified_g_Burkholderia (19.10%), unclassified_g_Weissella (10.75%), Weissella cibaria (7.78%), Pantoea dispersa (7.27%), Pantoea_sp._BK028 (4.10%), unclassified_g_Pantoea (3.59%), Pantoea rwandensis (3.36%), unclassified_f_Enterobacteriaceae (1.59%), Leuconostoc citreum (1.58%)*	*unclassified_g_Weissella (36.32%), Weissella cibaria (33.36%), Leuconostoc lactis (5.03%), Aspergillus phoenicis (3.18%), Saccharomyces cerevisiae (2.98%), Aspergillus niger (2.53%), Kosakonia cowanii (2.35%), Burkholderia gladioli (2.33%), unclassified_g_Burkholderia (1.90%), Aspergillus carbonarius (1.72%)， unclassified_f_Enterobacteriaceae (1.71%)*

	3-hydroxyacyl-[acyl-carrier-protein] dehydratase	4.2.1.59	*unclassified_g_Weissella (33.79%), Weissella cibaria (28.90%), unclassified_g_Burkholderia (19.72%), unclassified_g_Pantoea (9.30%), Enterobacter hormaechei (2.76%), unclassified_g_Leuconostoc (1.44%), Citrobacter koseri (1.33%), Leuconostoc lactis (1.04%)*	*unclassified_g_Weissella (46.92%), Weissella cibaria (38.68%), Leuconostoc lactis (4.45%), unclassified_g_Leuconostoc (4.25%), Enterobacter hormaechei (2.93%)*
	enoyl-[acyl-carrier-protein] reductase (NADPH, Si-specific)	1.3.1.10	*Enterobacter hormaechei (42.68%), Klebsiella pneumoniae (19.13%), Leuconostoc citreum (14.81%), unclassified_g_Pantoea (10.59%), unclassified_g_Lactococcus (7.32%), Enterobacter_sp._FS01 (4.53%)*	*Enterobacter hormaechei (79.36%), Leuconostoc citreum (5.50%), unclassified_g_Lactococcus (4.94%), unclassified_g_Bacillus_f_Bacillaceae (3.27%), unclassified_g_Pantoea (2.23%), Enterobacter_sp._FS01 (1.83%), Lactococcus garvieae (1.35%)*
	enoyl-[acyl-carrier-protein] reductase (NADH)	1.3.1.9	*unclassified_g_Burkholderia (57.84%), Enterobacter hormaechei (8.20%), Leuconostoc citreum (6.81%), Weissella confusa (6.10%), Enterobacter asburiae (5.98%), Klebsiella pneumoniae (3.67%), unclassified_f_Enterobacteriaceae (3.17%), unclassified_g_Pantoea (2.03%), unclassified_g_Leuconostoc (1.89%), Lactococcus lactis (1.42%), unclassified_g_Lactococcus (1.41%)*	*Enterobacter hormaechei (26.82%), Weissella confusa (22.08%), Enterobacter asburiae (20.85%), unclassified_g_Leuconostoc (13.39%), Leuconostoc citreum (4.85%), unclassified_g_Burkholderia (3.31%), Lactococcus lactis (2.07%), unclassified_g_Lactococcus (1.67%), unclassified_g_Bacillus_f_Bacillaceae (1.10%)*
	enoyl-[acyl-carrier-protein] reductase (NADPH)	1.3.1.104	-	*Bacillus amyloliquefaciens (50.00%), Bacillus nakamurai (50.00%)*
	trans-2-enoyl-CoA reductase (NAD+)	1.3.1.44	*unclassified_g_Burkholderia (99.97%)*	*unclassified_g_Burkholderia (90.44%), Burkholderia cepacia (7.35%), Variovorax_sp._SCN_67-85 (2.21%)*

	dodecanoyl-[acyl-carrier-protein] hydrolase	3.1.2.21	*unclassified_g_Weissella (91.57%), Leuconostoc citreum (3.74%), Leuconostoc lactis (3.46%)*	*unclassified_g_Weissella (91.48%), Leuconostoc lactis (7.32%)*
Higher alcohols biosynthesis	aspartate transaminase	2.6.1.1	*Burkholderia gladioli (45.69%), unclassified_g_Pantoea (21.44%), Type-C_symbiont_of_Plautia_stali (16.00%), Pantoea dispersa (5.18%), unclassified_g_Enterobacter (3.55%), unclassified_g_Kosakonia (3.03%), Saccharomyces cerevisiae (1.19%), Enterobacter cloacae (1.05%)*	*Saccharomyces cerevisiae (28.08%), Aspergillus luchuensis (21.82%), Aspergillus neoniger (18.17%), unclassified_g_Enterobacter (7.57%), unclassified_g_Kosakonia (6.43%), Monascus purpureus (4.42%), Burkholderia gladioli (2.92%), Bacillus amyloliquefaciens (2.08%), Aspergillus eucalypticola (2.03%), Shigella sonnei (1.79%), Enterobacter cloacae (1.35%), Enterobacter hormaechei (1.31%)*
	histidinol-phosphate transaminase	2.6.1.9	*Burkholderia gladioli (71.25%), unclassified_g_Pantoea (15.54%), unclassified_f_Enterobacteriaceae (2.76%), Leuconostoc citreum (2.47%), Leuconostoc lactis (2.32%), Escherichia coli (1.47%)*	*Aspergillus phoenicis (22.37%), Leuconostoc lactis (20.80%), Saccharomyces cerevisiae (18.94%), Burkholderia gladioli (10.53%), Escherichia coli (5.23%), unclassified_g_Burkholderia (4.26%), Enterobacter cloacae (2.74%), Leuconostoc citreum (2.45%), Enterobacter bugandensis (2.08%), unclassified_g_Enterobacter (1.68%), unclassified_g_Pantoea (1.47%), Bacillus amyloliquefaciens (1.29%), Enterobacter asburiae (1.19%), Burkholderia_sp._Bp8992 (1.08%), Pantoea deleyi (1.06%), Monascus purpureus (1.00%)*
	tyrosine transaminase	2.6.1.5	*Saccharomyces cerevisiae (82.63%), Monascus purpureus (11.80%), Aspergillus awamori (4.75%)*	*Monascus purpureus (56.04%), Saccharomyces cerevisiae (42.25%), Aspergillus niger (1.09%)*
	aromatic-amino-acid transaminase	2.6.1.57	Burkholderia gladioli (36.12%), unclassified_g_Burkholderia (34.04%),	*Monascus purpureus (33.55%), Saccharomyces cerevisiae (25.29%),*

			Pantoea_sp._Ap-959 (17.60%), Klebsiella pneumoniae (2.80%), Klebsiella quasipneumoniae (2.36%), unclassified_g_Enterobacter (1.56%)	*unclassified_g_Burkholderia (12.94%), Kosakonia cowanii (4.98%), unclassified_g_Enterobacter (4.54%), Enterobacteriaceae_bacterium_RIT693 (4.25%), Enterobacter asburiae (3.11%), Pantoea ananatis (2.32%), Enterobacter cloacae (2.13%), Cedecea lapagei (2.01%), Pantoea_sp. (1.66%)*
	phenylalanine (histidine) transaminase	2.6.1.58	*Saccharomyces cerevisiae (98.57%), [Candida] glabrata (1.43%)Saccharomyces cerevisiae (83.33%), Monascus purpureus (16.67%)*	*Saccharomyces cerevisiae (99.92%)*
	Carboxy-lyases	4.1.1.-	*Pantoea_sp._BK028 (35.38%), unclassified_f_Enterobacteriaceae (16.61%), Enterobacter roggenkampii (12.32%), unclassified_g_Enterobacter (12.04%), unclassified_g_Cronobacter (5.18%), Enterobacter ludwigii (3.79%), Klebsiella pneumoniae (3.32%), Lactococcus lactis (2.74%), Saccharomyces cerevisiae (1.97%), Enterobacter asburiae (1.19%), Franconibacter pulveris (1.14%)*	*Saccharomyces cerevisiae (37.38%), unclassified_g_Enterobacter (17.61%), Enterobacter roggenkampii (15.53%), unclassified_f_Enterobacteriaceae (8.56%), Enterobacter ludwigii (4.43%), unclassified_g_Cronobacter (3.17%), unclassified_g_Bacillus_f_Bacillaceae (2.18%), Lactococcus lactis (2.02%), Pantoea deleyi (1.65%), Enterobacter asburiae (1.32%)*
	pyruvate decarboxylase	4.1.1.1	*Saccharomyces cerevisiae (65.66%), Monascus purpureus (24.59%), Aspergillus piperis (6.26%), Penicillium coprophilum (1.86%), Rhizopus delemar (1.62%)*	*Saccharomyces cerevisiae (58.79%), Aspergillus niger (22.01%), Aspergillus piperis (16.20%), Monascus purpureus (2.23%)*
	phenylacetaldehyde dehydrogenase	1.2.1.39	*Klebsiella pneumoniae (40.15%), unclassified_g_Klebsiella (27.22%), unclassified_g_Enterobacter (23.23%), unclassified_o_Enterobacterales (9.32%)*	*unclassified_g_Enterobacter (65.05%), unclassified_o_Enterobacterales (28.24%), Burkholderia cepacia (2.65%), Burkholderia ambifaria (1.72%)*

	aldehyde dehydrogenase [NAD (P)+]	1.2.1.5	*Saccharomyces cerevisiae (78.82%), Monascus purpureus (20.00%), Aspergillus phoenicis (1.18%)*	*Saccharomyces cerevisiae (48.32%), Aspergillus eucalypticola (26.19%), Aspergillus phoenicis (22.68%), Monascus purpureus (2.77%)*
	aldehyde dehydrogenase (NAD+)	1.2.1.3	*Burkholderia gladioli (52.73%), unclassified_g_Burkholderia (40.32%), Pantoea dispersa (2.45%), Enterobacter asburiae (1.09%)*	*Saccharomyces cerevisiae (45.01%), Aspergillus niger (24.84%), Aspergillus phoenicis (7.99%), Enterobacter asburiae (5.16%), unclassified_g_Burkholderia (4.22%), Burkholderia gladioli (3.29%)*
	With NAD+ or NADP+ as acceptor	1.2.1.-	*Burkholderia gladioli (35.22%), unclassified_g_Burkholderia (30.03%), Pantoea dispersa (12.46%), unclassified_g_Pantoea (10.67%), Pseudomonas aeruginosa (5.67%), unclassified_g_Klebsiella (2.33%), Enterobacter roggenkampii (1.29%), unclassified_g_Enterobacter (1.03%)*	*Burkholderia gladioli (30.64%), Enterobacter roggenkampii (21.86%), Kosakonia_sp._CCTCC_M2018092 (10.51%), unclassified_g_Enterobacter (10.21%), unclassified_g_Burkholderia (9.53%), Pseudomonas aeruginosa (4.31%), Pantoea ananatis (2.80%), Burkholderia arboris (1.66%), Burkholderia cepacia (1.66%), unclassified_g_Pantoea (1.59%), Klebsiella pneumoniae (1.13%)*
	aryl-alcohol dehydrogenase	1.1.1.90	*unclassified_f_Enterobacteriaceae (99.19%)*	*unclassified_g_Burkholderia (47.50%)，Burkholderia cenocepacia (15.00%)，unclassified_f_Enterobacteriaceae (12.50%)，unclassified_g_Lactobacillus (12.50%)，Rhodococcus_sp._YH3-3 (7.50%)，Pediococcus pentosaceus (5.00%)*

	alcohol dehydrogenase	1.1.1.1	*Weissella cibaria (27.61%), Pediococcus pentosaceus (23.09%), Burkholderia gladioli (17.60%), Weissella_sp._DD23 (11.46%), Achromobacter xylosoxidans (5.84%), unclassified_g_Pantoea (4.29%), Klebsiella pneumoniae (1.91%), Weissella confusa (1.02%), Leuconostoc citreum (1.00%)*	*Weissella cibaria (39.44%), Pediococcus pentosaceus (23.71%), Weissella_sp._DD23 (15.40%), Saccharomyces cerevisiae (6.64%), Aspergillus niger (4.29%), unclassified_g_Leuconostoc (1.35%), Kosakonia cowanii (1.11%), Enterobacter asburiae (1.09%)*
	alcohol dehydrogenase (NADP+)	1.1.1.2	*unclassified_g_Burkholderia (39.88%), Mixta calida (19.51%), Pantoea dispersa (16.21%), unclassified_f_Enterobacteriaceae (10.71%), Klebsiella pneumoniae (3.51%), unclassified_g_Enterobacter (3.09%), Klebsiella variicola (1.23%)*	*unclassified_f_Enterobacteriaceae (22.81%), Vanderwaltozyma polyspora (20.97%), Aspergillus piperis (20.37%), unclassified_g_Enterobacter (12.49%), Saccharomyces cerevisiae (8.82%), Enterobacter ludwigii (2.83%), unclassified_g_Pantoea (2.42%)*
	acetolactate synthase	2.2.1.6	*unclassified_g_Burkholderia (24.53%), Burkholderia gladioli (21.18%), Weissella cibaria (20.32%), unclassified_g_Pantoea (15.16%), unclassified_f_Enterobacteriaceae (3.54%), Kosakonia cowanii (2.31%), Klebsiella pneumoniae (1.99%), unclassified_g_Enterobacter (1.61%), Enterobacter cloacae (1.47%), unclassified_d_Bacteria (1.34%)*	*Weissella cibaria (57.42%), Saccharomyces cerevisiae (5.32%), Kosakonia cowanii (5.29%), Aspergillus niger (4.81%), Leuconostoc lactis (4.60%), unclassified_g_Enterobacter (4.25%), Enterobacter cloacae (2.56%), unclassified_f_Enterobacteriaceae (2.19%), unclassified_o_Enterobacterales (1.82%)*
	ketol-acid reductoisomerase (NADP+)	1.1.1.86	*unclassified_g_Burkholderia (67.43%), Kosakonia cowanii (10.12%), Enterobacter hormaechei (5.52%), Pantoea_sp._ARC607 (4.38%), Pantoea ananatis (3.18%), Pantoea agglomerans (1.77%), Lactococcus lactis (1.58%), Klebsiella pneumoniae (1.46%), unclassified_g_Cronobacter (1.12%)*	*Saccharomyces cerevisiae (29.46%), Kosakonia cowanii (21.28%), Aspergillus awamori (19.55%), Enterobacter hormaechei (5.75%), Pantoea_sp._ARC607 (5.53%), Pantoea agglomerans (3.80%), unclassified_g_Burkholderia (3.66%), Klebsiella pneumoniae (2.48%), Escherichia coli (1.82%)*

	dihydroxy-acid dehydratase	4.2.1.9	*Burkholderia gladioli (52.92%), Burkholderia_sp._SJZ089 (24.16%), Pantoea_sp._R102 (8.87%), unclassified_d_Bacteria (3.92%), Enterobacter cloacae (2.59%), Escherichia coli (1.16%)*	*Aspergillus niger (43.07%), Monascus purpureus (20.12%), Saccharomyces cerevisiae (9.01%), Enterobacter cloacae (6.22%), Aspergillus flavus (4.16%), Burkholderia gladioli (3.50%), Salmonella enterica (3.04%)*
	branched-chain-amino-acid transaminase	2.6.1.42	*Burkholderia gladioli (47.63%), Weissella cibaria (43.33%), unclassified_d_Bacteria (3.03%), unclassified_g_Pantoea (1.40%), Leuconostoc citreum (1.17%),*	*Weissella cibaria (67.99%), Aspergillus niger (13.31%), Saccharomyces cerevisiae (8.74%), Aspergillus awamori (3.26%), Burkholderia gladioli (1.29%), unclassified_o_Enterobacterales (1.01%)*
	valine dehydrogenase (NAD+)	1.4.1.23	*Rhodococcus qingshengii (100.00%)*	*Rhodococcus qingshengii (100.00%)*
	valine---pyruvate transaminase	2.6.1.66	*Burkholderia gladioli (33.38%), Enterobacter ludwigii (23.20%), Pantoea dispersa (16.76%), unclassified_f_Enterobacteriaceae (10.76%), Burkholderia_sp._Tr-862 (7.35%), Enterobacter asburiae (1.66%), Lactococcus lactis (1.53%)*	*unclassified_f_Enterobacteriaceae (32.83%), Enterobacter asburiae (16.51%), Enterobacter ludwigii (16.40%), Burkholderia gladioli (4.13%), Lactococcus lactis (3.67%), Pantoea_sp._ARC607 (3.36%), Pantoea ananatis (3.24%), unclassified_f_Erwiniaceae (3.16%), Kosakonia cowanii (3.13%), Enterobacter_sp._Acro-832 (2.55%)*
	2-isopropylmalate synthase	2.3.3.13	*unclassified_g_Burkholderia (57.23%), Pantoea dispersa (12.36%), unclassified_g_Pantoea (12.18%), unclassified_f_Enterobacteriaceae (5.65%), Leuconostoc citreum (1.97%), Klebsiella pneumoniae (1.91%), Kosakonia cowanii (1.62%), Enterobacter cloacae (1.38%)*	*Saccharomyces cerevisiae (37.18%), Aspergillus niger (14.61%), unclassified_f_Enterobacteriaceae (10.76%), Kosakonia cowanii (6.74%), unclassified_g_Burkholderia (5.34%), Enterobacter roggenkampii (2.98%), Enterobacter cloacae (2.87%), Enterobacter hormaechei (2.36%), unclassified_g_Enterobacter (2.01%)*

	3-isopropylmalate dehydratase	4.2.1.33	*Burkholderia gladioli (49.67%), unclassified_g_Burkholderia (15.36%), unclassified_g_Pantoea (13.52%), Pantoea dispersa (9.75%), Leuconostoc citreum (2.66%), unclassified_f_Enterobacteriaceae (2.59%), unclassified_g_Enterobacter (1.53%)*	*Saccharomyces cerevisiae (24.29%), Aspergillus niger (17.72%), unclassified_f_Enterobacteriaceae (15.08%), unclassified_g_Enterobacter (10.22%), Burkholderia gladioli (6.40%), Leuconostoc citreum (4.50%), Escherichia coli (3.90%), unclassified_g_Pantoea (3.15%), unclassified_g_Burkholderia (2.46%)*
	3-isopropylmalate dehydrogenase	1.1.1.85	*Burkholderia gladioli (53.17%), unclassified_g_Pantoea (26.64%), Kosakonia_sp._CCTCC_M2018092 (5.43%), Leuconostoc citreum (4.40%), unclassified_f_Enterobacteriaceae (1.89%), Enterobacter bugandensis (1.54%), Klebsiella pneumoniae (1.25%), Lactococcus lactis (1.15%)*	*Aspergillus niger (21.18%), Saccharomyces cerevisiae (20.93%), Kosakonia_sp._CCTCC_M2018092 (15.31%), Monascus purpureus (14.91%), unclassified_f_Enterobacteriaceae (5.41%), Burkholderia gladioli (3.88%), Leuconostoc citreum (3.48%), Enterobacter bugandensis (2.03%), Klebsiella variicola (2.03%), unclassified_g_Enterobacter (2.01%)*
	leucine dehydrogenase	1.4.1.9	*unclassified_g_Rhodococcus_f_Nocardiaceae (100.00%)*	*Bacillus amyloliquefaciens (93.88%), unclassified_g_Rhodococcus_f_Nocardiaceae (6.12%)*
	methylglyoxal reductase (NADPH)	1.1.1.283	*Saccharomyces cerevisiae (63.89%), Lactococcus lactis (35.61%)*	*Saccharomyces cerevisiae (96.32%), Lactococcus lactis (1.91%), Bacillus amyloliquefaciens (1.06%)*
Valine and leucine biosynthesis	acetolactate synthase	2.2.1.6	*unclassified_g_Burkholderia (24.53%), Burkholderia gladioli (21.18%), Weissella cibaria (20.32%), unclassified_g_Pantoea (15.16%), unclassified_f_Enterobacteriaceae (3.54%), Kosakonia cowanii (2.31%), Klebsiella pneumoniae (1.99%), unclassified_g_Enterobacter (1.61%),*	*Weissella cibaria (57.42%), Saccharomyces cerevisiae (5.32%), Kosakonia cowanii (5.29%), Aspergillus niger (4.81%), Leuconostoc lactis (4.60%), unclassified_g_Enterobacter (4.25%), Enterobacter cloacae (2.56%), unclassified_f_Enterobacteriaceae (2.19%), unclassified_o_Enterobacterales (1.82%)*

			Enterobacter cloacae (1.47%), unclassified_d_Bacteria (1.34%)	
	ketol-acid reductoisomerase (NADP+)	1.1.1.86	*unclassified_g_Burkholderia (67.43%), Kosakonia cowanii (10.12%), Enterobacter hormaechei (5.52%), Pantoea_sp._ARC607 (4.38%), Pantoea ananatis (3.18%), Pantoea_agglomerans (1.77%), Lactococcus lactis (1.58%), Klebsiella pneumoniae (1.46%), unclassified_g_Cronobacter (1.12%)*	*Saccharomyces cerevisiae (29.46%), Kosakonia cowanii (21.28%), Aspergillus awamori (19.55%), Enterobacter hormaechei (5.75%), Pantoea_sp._ARC607 (5.53%), Pantoea agglomerans (3.80%), unclassified_g_Burkholderia (3.66%), Klebsiella pneumoniae (2.48%), Escherichia coli (1.82%)*
	dihydroxy-acid dehydratase	4.2.1.9	*Burkholderia gladioli (52.92%), Burkholderia_sp._SJZ089 (24.16%), Pantoea_sp._R102 (8.87%), unclassified_d_Bacteria (3.92%), Enterobacter cloacae (2.59%), Escherichia coli (1.16%)*	*Aspergillus niger (43.07%), Monascus purpureus (20.12%), Saccharomyces cerevisiae (9.01%), Enterobacter cloacae (6.22%), Aspergillus flavus (4.16%), Burkholderia gladioli (3.50%), Salmonella enterica (3.04%)*
	2-isopropylmalate synthase	2.3.3.13	*unclassified_g_Burkholderia (57.23%), Pantoea dispersa (12.36%), unclassified_g_Pantoea (12.18%), unclassified_f_Enterobacteriaceae (5.65%), Leuconostoc citreum (1.97%), Klebsiella pneumoniae (1.91%), Kosakonia cowanii (1.62%), Enterobacter cloacae (1.38%)*	*Saccharomyces cerevisiae (37.18%), Aspergillus niger (14.61%), unclassified_f_Enterobacteriaceae (10.76%), Kosakonia cowanii (6.74%), unclassified_g_Burkholderia (5.34%), Enterobacter roggenkampii (2.98%), Enterobacter cloacae (2.87%), Enterobacter hormaechei (2.36%), unclassified_g_Enterobacter (2.01%)*

	3-isopropylmalate dehydratase	4.2.1.33	*Burkholderia gladioli (49.67%), unclassified_g_Burkholderia (15.36%), unclassified_g_Pantoea (13.52%), Pantoea dispersa (9.75%), Leuconostoc citreum (2.66%), unclassified_f_Enterobacteriaceae (2.59%), unclassified_g_Enterobacter (1.53%)*	*Saccharomyces cerevisiae (24.29%), Aspergillus niger (17.72%), unclassified_f_Enterobacteriaceae (15.08%), unclassified_g_Enterobacter (10.22%), Burkholderia gladioli (6.40%), Leuconostoc citreum (4.50%), Escherichia coli (3.90%), unclassified_g_Pantoea (3.15%), unclassified_g_Burkholderia (2.46%)*
	3-isopropylmalate dehydrogenase	1.1.1.85	*Burkholderia gladioli (53.17%), unclassified_g_Pantoea (26.64%), Kosakonia_sp._CCTCC_M2018092 (5.43%), Leuconostoc citreum (4.40%), unclassified_f_Enterobacteriaceae (1.89%), Enterobacter bugandensis (1.54%), Klebsiella pneumoniae (1.25%), Lactococcus lactis (1.15%)*	*Aspergillus niger (21.18%), Saccharomyces cerevisiae (20.93%), Kosakonia_sp._CCTCC_M2018092 (15.31%), Monascus purpureus (14.91%), unclassified_f_Enterobacteriaceae (5.41%), Burkholderia gladioli (3.88%), Leuconostoc citreum (3.48%), Enterobacter bugandensis (2.03%), Klebsiella variicola (2.03%), unclassified_g_Enterobacter (2.01%)*
	branched-chain-amino-acid transaminase	2.6.1.42	*Burkholderia gladioli (47.63%), Weissella cibaria (43.33%), unclassified_d_Bacteria (3.03%), unclassified_g_Pantoea (1.40%), Leuconostoc citreum (1.17%),*	*Weissella cibaria (67.99%), Aspergillus niger (13.31%), Saccharomyces cerevisiae (8.74%), Aspergillus awamori (3.26%), Burkholderia gladioli (1.29%), unclassified_o_Enterobacterales (1.01%)*
	valine---pyruvate transaminase	2.6.1.66	*Burkholderia gladioli (33.38%), Enterobacter ludwigii (23.20%), Pantoea dispersa (16.76%), unclassified_f_Enterobacteriaceae (10.76%), Burkholderia_sp._Tr-862 (7.35%), Enterobacter asburiae (1.66%), Lactococcus lactis (1.53%)*	*unclassified_f_Enterobacteriaceae (32.83%), Enterobacter asburiae (16.51%), Enterobacter ludwigii (16.40%), Burkholderia gladioli (4.13%), Lactococcus lactis (3.67%), Pantoea_sp._ARC607 (3.36%), Pantoea ananatis (3.24%), unclassified_f_Erwiniaceae (3.16%), Kosakonia cowanii (3.13%), Enterobacter_sp._Acro-832 (2.55%)*

	leucine dehydrogenase	1.4.1.9	*unclassified_g_Rhodococcus_f_Nocardiaceae (100.00%)*	*Bacillus amyloliquefaciens (93.88%), unclassified_g_Rhodococcus_f_Nocardiaceae (6.12%)*
Phenylalanine biosynthesis	chorismate mutase	5.4.99.5	*Burkholderia gladioli (50.24%), unclassified_g_Pantoea (13.65%), Pantoea_sp._GL120224-02 (11.78%), Pantoea dispersa (8.34%), Kosakonia cowanii (2.86%), unclassified_f_Enterobacteriaceae (2.76%), Klebsiella pneumoniae (1.75%), Enterobacter cloacae (1.63%), Enterobacter kobei (1.16%), Klebsiella variicola (1.01%)*	*Saccharomyces cerevisiae (18.24%), Kosakonia cowanii (14%), Aspergillus niger (13.34%), Burkholderia gladioli (11.06%), Enterobacter cloacae (9.63%), Enterobacter kobei (7.73%), unclassified_g_Enterobacter (3.67%), Pantoea ananatis (3.05%),Pantoea deleyi (2.95%), Bacillus amyloliquefaciens (2.76%), unclassified_g_Pantoea (2.22%), Enterobacter asburiae (2.07%), Enterobacter quasihormaechei (1.61%), Lactococcus lactis (1.26%), Enterobacter oligotrophica (1.03%)*
	arogenate dehydratase	4.2.1.91	*Burkholderia gladioli (60.93%), Pantoea dispersa (30.17%), Klebsiella pneumoniae (7.12%), Pantoea ananatis (1.07%)*	*Burkholderia gladioli (52.11%), Pantoea agglomerans (24.16%), Pantoea ananatis (17.05%), Burkholderia seminalis (4.16%), Klebsiella pneumoniae (1.96%)*
	prephenate dehydratase	4.2.1.51	*Burkholderia gladioli (59.83%), Pantoea_sp._GL120224-02 (13.26%), Pantoea dispersa (13.02%), unclassified_f_Enterobacteriaceae (3.10%), Klebsiella pneumoniae (3.07%), Kosakonia cowanii (1.68%), Enterobacter cloacae (1.09%)*	*Saccharomyces cerevisiae (39.04%), Burkholderia gladioli (13.42%), unclassified_f_Enterobacteriaceae (9.99%), Enterobacter cloacae (7.95%), Monascus purpureus (7.54%), unclassified_g_Enterobacter (4.72%), Pantoea ananatis (3.84%), Pantoea deleyi (2.60%), Pantoea agglomerans (2.53%), Enterobacter quasihormaechei (2.07%), Lactococcus lactis (1.68%), unclassified_g_Burkholderia (1.26%), Bacillus amyloliquefaciens (1.17%)*

	aspartate transaminase	2.6.1.1	*Burkholderia gladioli (45.69%), unclassified_g_Pantoea (21.44%), Type-C_symbiont_of_Plautia_stali (16.00%), Pantoea dispersa (5.18%), unclassified_g_Enterobacter (3.55%), unclassified_g_Kosakonia (3.03%), Saccharomyces cerevisiae (1.19%), Enterobacter cloacae (1.05%)*	*Saccharomyces cerevisiae (28.08%), Aspergillus luchuensis (21.82%), Aspergillus neoniger (18.17%), unclassified_g_Enterobacter (7.57%), unclassified_g_Kosakonia (6.43%), Monascus purpureus (4.42%), Burkholderia gladioli (2.92%), Bacillus amyloliquefaciens (2.08%), Aspergillus eucalypticola (2.03%), Shigella sonnei (1.79%), Enterobacter cloacae (1.35%), Enterobacter hormaechei (1.31%)*
	histidinol-phosphate transaminase	2.6.1.9	*Burkholderia gladioli (71.25%), unclassified_g_Pantoea (15.54%), unclassified_f_Enterobacteriaceae (2.76%), Leuconostoc citreum (2.47%), Leuconostoc lactis (2.32%), Escherichia coli (1.47%)*	*Aspergillus phoenicis (22.37%), Leuconostoc lactis (20.80%), Saccharomyces cerevisiae (18.94%), Burkholderia gladioli (10.53%), Escherichia coli (5.23%), unclassified_g_Burkholderia (4.26%), Enterobacter cloacae (2.74%), Leuconostoc citreum (2.45%), Enterobacter bugandensis (2.08%), unclassified_g_Enterobacter (1.68%), unclassified_g_Pantoea (1.47%), Bacillus amyloliquefaciens (1.29%), Enterobacter asburiae (1.19%), Burkholderia_sp._Bp8992 (1.08%), Pantoea deleyi (1.06%), Monascus purpureus (1.00%)*
	phenylalanine (histidine) transaminase	2.6.1.58	*Saccharomyces cerevisiae (98.57%), [Candida] glabrata (1.43%)Saccharomyces cerevisiae (83.33%), Monascus purpureus (16.67%)*	*Saccharomyces cerevisiae (99.92%)*
	aromatic-amino-acid transaminase	2.6.1.57	*Burkholderia gladioli (36.12%), unclassified_g_Burkholderia (34.04%), Pantoea_sp._Ap-959 (17.60%), Klebsiella pneumoniae (2.80%), Klebsiella*	*Monascus purpureus (33.55%), Saccharomyces cerevisiae (25.29%), unclassified_g_Burkholderia (12.94%), Kosakonia cowanii (4.98%),*

			quasipneumoniae (2.36%), unclassified_g_Enterobacter (1.56%)	unclassified_g_Enterobacter (4.54%), Enterobacteriaceae_bacterium_RIT693 (4.25%), Enterobacter asburiae (3.11%), Pantoea ananatis (2.32%), Enterobacter cloacae (2.13%), Cedecea lapagei (2.01%), Pantoea_sp. (1.66%)
	tyrosine transaminase	2.6.1.5	*Saccharomyces cerevisiae (82.63%), Monascus purpureus (11.80%), Aspergillus awamori (4.75%)*	*Monascus purpureus (56.04%), Saccharomyces cerevisiae (42.25%), Aspergillus niger (1.09%)*
Esters biosynthesis	carboxylesterase	3.1.1.1	*unclassified_g_Burkholderia (51.96%), Burkholderia gladioli (47.98%)*	*Aspergillus niger (83.26%), Burkholderia gladioli (6.01%), unclassified_g_Burkholderia (5.54%), unclassified_g_Bacillus_f_Bacillaceae (2.68%), Bacillus ginsengihumi (1.63%)*
	triacylglycerol lipase	3.1.1.3	*Burkholderia gladioli (81.08%), unclassified_o_Enterobacterales (8.19%), Saccharomyces cerevisiae (5.70%), Enterobacter cloacae (3.40%), Monascus purpureus (1.49%)*	*Saccharomyces cerevisiae (61.93%), Aspergillus niger (16.09%), Aspergillus phoenicis (12.99%), Enterobacter cloacae (4.65%), Monascus purpureus (1.75%), Burkholderia gladioli (1.49%)*
	alcohol O-acetyltransferase	2.3.1.84	*Saccharomyces cerevisiae (100.00%)*	*Saccharomyces cerevisiae (100.00%)*
	alcohol dehydrogenase	1.1.1.1	*Weissella cibaria (27.61%), Pediococcus pentosaceus (23.09%), Burkholderia gladioli (17.60%), Weissella_sp._DD23 (11.46%), Achromobacter xylosoxidans (5.84%), unclassified_g_Pantoea (4.29%), Klebsiella pneumoniae (1.91%), Weissella confusa (1.02%), Leuconostoc citreum (1.00%)*	*Weissella cibaria (39.44%), Pediococcus pentosaceus (23.71%), Weissella_sp._DD23 (15.40%), Saccharomyces cerevisiae (6.64%), Aspergillus niger (4.29%), unclassified_g_Leuconostoc (1.35%), Kosakonia cowanii (1.11%), Enterobacter asburiae (1.09%)*
2-Methoxy-4-vinylphenol biosynthesis	ferulic acid decarboxylase	4.1.1.102	*Saccharomyces cerevisiae (54.00%), Monascus purpureus (46.00%)*	*Monascus purpureus (58.62%), Saccharomyces cerevisiae (41.28%)*
